# Supplementary material for: Potassium nutrient response in the rice-wheat cropping system in different agro-ecozones of Nepal
Source: PLoS One. 2021 Mar 18;16(3):e0248837. doi: 10.1371/journal.pone.0248837 (PMC7971854; doi:10.1371/journal.pone.0248837)
Supplement: S1 File — (DOCX) [file pone.0248837.s001.docx]

**Title**

Potassium nutrient response in rice-wheat cropping system in different agro-ecozones of Nepal

**S1 Table.** Mean soil chemical properties after first year of study as affected by the different rates of potassium fertilizer at different research sites of Nepal.

| Rate  kg K_2_O ha^-1^ | pH |  | OC | N |  | P_2_O_5_ | K_2_O |
| --- | --- | --- | --- | --- | --- | --- | --- |
|  |  |  | % | |  | kg ha^-1^ | |
| *Location: Kabre, Dolakha (2012)* | | | | | | | |
| 0 | 4.4 |  | 1.04 | 0.09 |  | 97.21 | 141.87 b^†^ |
| 15 | 4.4 |  | 1.05 | 0.07 |  | 121.05 | 281.81 a |
| 30 | 4.3 |  | 1.17 | 0.09 |  | 120.21 | 283.83 a |
| 45 | 4.2 |  | 1.14 | 0.10 |  | 180.32 | 288.84 a |
| 60 | 4.3 |  | 1.09 | 0.16 |  | 100.21 | 309.55 a |
| 75 | 4.2 |  | 1.22 | 0.11 |  | 197.21 | 292.20 a |
| 90 | 4.2 |  | 1.24 | 0.15 |  | 161.28 | 306.51 a |
| Significance | NS |  | NS | NS |  | NS | ** |
| *Location: Rampur, Chitwan (2010)* | | | | | | | |
| 0 | 5.6 |  | 1.39 | 0.15 |  | 212.91 c | 122.55 c |
| 15 | 5.6 |  | 1.80 | 0.20 |  | 218.11 c | 118.81 c |
| 30 | 5.4 |  | 1.52 | 0.17 |  | 243.67 ab | 118.75 c |
| 45 | 5.5 |  | 1.78 | 0.20 |  | 217.80 c | 126.25 c |
| 60 | 5.5 |  | 1.69 | 0.19 |  | 214.45 c | 152.75 b |
| 75 | 5.5 |  | 1.72 | 0.19 |  | 261.67 a | 158.75 b |
| 90 | 5.5 |  | 1.64 | 0.19 |  | 224.30 bc | 204.75 a |
| Significance | NS |  | NS | NS |  | ** | * |
| *Location: Parwanipur, Bara (2013)* | | | | | | | |
| 0 | 5.9 |  | 0.64 | 0.09 |  | 74.31 | 224.00 |
| 15 | 5.6 |  | 0.68 | 0.08 |  | 110.77 | 225.33 |
| 30 | 5.7 |  | 0.71 | 0.11 |  | 108.42 | 230.67 |
| 45 | 5.6 |  | 0.78 | 0.12 |  | 112.69 | 232.00 |
| 60 | 5.5 |  | 0.74 | 0.10 |  | 116.22 | 229.33 |
| 75 | 5.5 |  | 0.85 | 0.14 |  | 114.02 | 242.67 |
| 90 | 5.4 |  | 0.77 | 0.13 |  | 118.89 | 246.67 |
| Significance | NS |  | NS | NS |  | NS | NS |

^†^Means for each variable followed by same lowercase letters are not significantly different.

****P* < 0.001, ***P* < 0.01, **P* < 0.05, NS = not significant.

**S2 Table.** Effect of different rates of potassium fertilizer on plant height, panicle length, and tiller number per square meter of rice in rice-wheat cropping system at Rampur, Chitwan in three consecutive years.

| Rate  kg K_2_O_5_ ha^-1^ | Plant height, cm | | | Tillers, m^-2^ | | | Panicle length, cm | | | |
| --- | --- | --- | --- | --- | --- | --- | --- | --- | --- | --- |
|  | 2009 | 2010 | 2011 | 2009 | 2010 | 2011 | 2009 | | 2010 | 2011 |
| 0 | 83.5^d†^ | 73.0^b^ | 64.9^d^ | 185.0^b^ | 187.0^b^ | 248.0^a^ | 20.5^b^ | | 21.2^b^ | 22.3^b^ |
| 15 | 98.0^bc^ | 84.5^ab^ | 80.8^c^ | 263.0^a^ | 243.0^ab^ | 312.0^a^ | 23.0^a^ | | 22.7^a^ | 24.0^a^ |
| 30 | 97.0^c^ | 82.0^ab^ | 84.2^bc^ | 262.0^a^ | 275.0^a^ | 264.0^a^ | 24.0^a^ | | 23.2^a^ | 23.2^ab^ |
| 45 | 102.0^ab^ | 84.0^ab^ | 88.0^ab^ | 267.0^a^ | 260.0^ab^ | 310.0^a^ | 23.6^a^ | | 22.5^a^ | 23.5^a^ |
| 60 | 100.5^abc^ | 88.0^a^ | 89.3^ab^ | 244.0^a^ | 262.0^ab^ | 321.0^a^ | 24.5^a^ | | 22.7^a^ | 23.1^ab^ |
| 75 | 104.5^a^ | 84.0^ab^ | 90.4^a^ | 267.0^a^ | 259.0^ab^ | 322.0^a^ | 23.6^a^ | | 22.2^a^ | 23.8^a^ |
| 90 | 103.0^a^ | 85.0^ab^ | 87.8^ab^ | 254.0^a^ | 237.0^ab^ | 248.0^a^ | 24.5^a^ | | 23.0^a^ | 24.0^a^ |
| Significance | ** | ** | ** | ** | ** | ** | ** | ** | | ** |

^†^Means for each variable followed by same lowercase letters are not significantly different.

***P* < 0.01

**S3 Table.** Correlation analysis of rice yield and yield attributing traits at Rampur, Chitwan, Nepal.

|  | Plant height | Panicle length | Tillers | Grain yield | Straw yield | Thousand grain weight |
| --- | --- | --- | --- | --- | --- | --- |
| Plant height | 1 |  |  |  |  |  |
| Panicle length | 0.532* | 1 |  |  |  |  |
| Tillers | 0.371 | 0.438 | 1 |  |  |  |
| Grain yield | 0.876** | 0.516 | 0.524 | 1 |  |  |
| Straw yield | 0.852** | 0.499 | 0.521 | 0.885** | 1 |  |
| Thousand grain weight | -0.047 | -0.061 | 0.277 | 0.127 | 0.062 | 1 |

****P* < 0.001, ***P* < 0.01, **P* < 0.05, NS = not significant.

**S4 Table.** Effect of different rates of potassium fertilizer on plant height, panicle length, and tiller number per square meter of wheat in rice-wheat cropping system at Rampur, Chitwan, Nepal in three consecutive years.

| Rate,  kg K_2_O_5_ ha^-1^ | Plant height, cm | | | Tillers, m^-2^ | | | Panicle Length, m | | |
| --- | --- | --- | --- | --- | --- | --- | --- | --- | --- |
|  | 2009 | 2010 | 2011 | 2009 | 2010 | 2011 | 2009 | 2010 | 2011 |
| 0 | 79.2e^†^ | 61.0e | 63.9d | 259.0b | 274.0 | 248.0 | 11.7b | 9.5c | 9.4b |
| 15 | 92.0d | 73.2d | 80.8c | 338.0a | 261.0 | 311.0 | 14.2a | 12.2b | 12.1a |
| 30 | 99.0bc | 79.7bc | 84.2bc | 360.0a | 252.0 | 263.0 | 14.5a | 13.2a | 12.9a |
| 45 | 97.7c | 78.7c | 88.0ab | 370.0a | 263.0 | 310.0 | 14.7a | 12.7ab | 12.2a |
| 60 | 100.5ab | 81.2abc | 89.3ab | 367.0a | 2810 | 320.0 | 15.0a | 13.0ab | 12.5a |
| 75 | 100.7ab | 84.7a | 90.4a | 376.0a | 313.0 | 321.0 | 14.7a | 13.0ab | 11.7a |
| 90 | 102.2a | 83.7ab | 87.8ab | 329.0ab | 252.0 | 260.0 | 15.0a | 13.0ab | 12.8a |
| Significance | ** | ** | ** | * | NS | NS | ** | ** | ** |

^†^Means for each variable followed by same lowercase letters are not significantly different.

***P* < 0.01, **P* < 0.05, NS = not significant.

**S5 Table.** Correlation analysis of wheat yield and yield attributing traits at Rampur, Chitwan, Nepal.

|  | Plant height | Panicle length | Tillers | Grain yield | Straw yield | Thousand  grain weight |
| --- | --- | --- | --- | --- | --- | --- |
| Plant height | 1 |  |  |  |  |  |
| Panicle length | 0.815** | 1 |  |  |  |  |
| Tillers | 0.552* | 0.357 | 1 |  |  |  |
| Grain yield | 0.884** | 0.743* | 0.506 | 1 |  |  |
| Straw yield | -0.194 | -0.292 | -0.224 | -0.127 | 1 |  |
| Thousand grain weight | 0.841** | 0.777* | 0.454 | 0.779* | -0.390 | 1 |

****P* < 0.001, ***P* < 0.01, **P* < 0.05, NS = not significant.

**S6 Table.** Effect of different rates of potassium fertilizer on plant height, tiller number and panicle length of rice and wheat in rice-wheat cropping system at Kabre, Dolakha, Nepal in two consecutive years.

| Rice | | | | | | | Wheat | | |
| --- | --- | --- | --- | --- | --- | --- | --- | --- | --- |
| Rate  kg K_2_O_5_ ha^-1^ | Plant  height, cm | | Tillers,  m^-2^ | | Panicle  length, cm | | Plant  height, cm | Tillers,  m^-2^ | Panicle  length, cm |
|  | 2010 | 2011 | 2010 | 2011 | 2010 | 2011 | 2011 | 2011 | 2011 |
| 0 | 85.6b | 84.9c | 190.0c | 137.0b | 19.7 | 20.3 | 68.5b | 147.0b | 7.4c |
| 15 | 101.8a | 96.8b | 295.0a | 216.0a | 20.7 | 19.7 | 80.5a | 223.0a | 9.6ab |
| 30 | 103.1a | 102.8ab | 286.0a | 221.0a | 21.2 | 20.6 | 78.1a | 246.0a | 9.2ab |
| 45 | 101.3a | 104.2a | 257.0b | 198.0a | 21.2 | 20.4 | 82.7a | 247.0a | 9.2ab |
| 60 | 100.0a | 100.8ab | 293.0a | 196.0a | 20.7 | 20.5 | 81.1a | 234.0a | 10.1a |
| 75 | 102.3a | 99.8ab | 250.0b | 228.0a | 21.4 | 20.6 | 79.4a | 233.0a | 9.6ab |
| 90 | 102.5a | 104.6a | 281.0a | 223.0a | 21.9 | 20.5 | 79.1a | 255.0a | 9.1b |
| P value | ** | ** | ** | ** | NS | NS | ** | ** | ** |

^†^Means for each variable followed by same lowercase letters are not significantly different.

***P* < 0.01, **P* < 0.05, NS = not significant.

**S7 Table.** Correlation analysis of rice yield and yield attributing traits at Kabre, Dolakha, Nepal.

|  | Plant height | Panicle length | Tiller | Thousand grain weight | Grain  yield | Straw yield |
| --- | --- | --- | --- | --- | --- | --- |
| Plant height | 1 |  |  |  |  |  |
| Panicle length | 0.401 | 1 |  |  |  |  |
| Tillers | 0.502 | 0.262 | 1 |  |  |  |
| Thousand grain weight | -0.284 | -0.208 | -0.444 | 1 |  |  |
| Grain yield | 0.805** | 0.241 | 0.496 | -0.370 | 1 |  |
| Straw yield | 0.636* | 0.288 | 0.808** | -0.315 | 0.626* | 1 |

****P* < 0.001, ***P* < 0.01, **P* < 0.05, NS = not significant.

**S8 Table.** Correlation analysis of wheat yield and yield attributing traits at Kabre, Dolakha, Nepal.

|  | Plant height | Panicle length | Tiller | Thousand grain weight | Grain yield | Straw yield |
| --- | --- | --- | --- | --- | --- | --- |
| Plant height | 1 |  |  |  |  |  |
| Panicle length | 0.462 | 1 |  |  |  |  |
| Tillers | 0.701* | 0.309 | 1 |  |  |  |
| Thousand grain weight | 0.214 | 0.186 | 0.213 | 1 |  |  |
| Grain yield | 0.682* | 0.383 | 0.640* | 0.165 | 1 |  |
| Straw yield | 0.752* | 0.503 | 0.694* | 0.280 | 0.824** | 1 |

****P* < 0.001, ***P* < 0.01, **P* < 0.05, NS = not significant.

**S9 Table.** Effect of different rates of potassium fertilizer on plant height, panicle length, and tiller numbers of rice and wheat in rice-wheat cropping system at Parwanipur, Bara, Nepal in 2012 and 2014.

| Rate  kg K_2_O_5_ ha^-1^ | Rice | | | | | | Wheat | | | | | |
| --- | --- | --- | --- | --- | --- | --- | --- | --- | --- | --- | --- | --- |
|  | Plant height,  cm | | Panicle length,  cm | | Tillers,  m^-2^ | | Plant height,  cm | | Panicle length,  cm | | Tillers,  m^-2^ | |
|  | 2012 | 2014 | 2012 | 2014 | 2012 | 2014 | 2013 | 2014 | 2013 | 2014 | 2013 | 2014 |
| 0 | 76.1 | 79.1b^†^ | 22.9c | 22.9b | 163.0c | 146.0b | 58.2c | 70.4b | 6.1b | 6.6b | 259.0 | 170.0b |
| 15 | 84.6 | 96.2a | 24.4ab | 25.8a | 224.0a | 244.0a | 82.6b | 83.2a | 8.8a | 8.1a | 282.0 | 242.0a |
| 30 | 84.8 | 95.9a | 24.8a | 25.7a | 212.0ab | 245.0a | 87.7a | 83.3a | 8.3a | 8.0a | 295.0 | 235.0a |
| 45 | 80.6 | 92.6a | 23.3bc | 25.4a | 182.0bc | 221.0a | 86.8a | 79.7a | 8.8a | 7.8a | 283.0 | 257.0a |
| 60 | 84.0 | 98.9a | 24.3ab | 26.8a | 218.0a | 242.0a | 89.2a | 85.1a | 8.9a | 8.3a | 242.0 | 236.0a |
| 75 | 81.6 | 95.8a | 24.0abc | 25.6a | 211.0ab | 232.0a | 88.4a | 85.7a | 8.7a | 8.1a | 284.0 | 261.0a |
| 90 | 82.6 | 99.4a | 24.5ab | 26.4a | 216.0a | 260.0a | 87.3a | 82.7a | 8.8a | 8.1a | 312.0 | 263.0a |
| P value | NS | *** | * | *** | ** | *** | *** | * | ** | ** | NS | ** |

^†^Means for each variable followed by same lowercase letters are not significantly different.

****P* < 0.001, ***P* < 0.01, **P* < 0.05, NS = not significant.

**S10 Table.** Correlation analysis of rice yield and yield attributing traits at Parwanipur, Bara, Nepal.

|  | Plant height | Panicle length | Tiller | Thousand grain weight | Grain yield | Straw yield |
| --- | --- | --- | --- | --- | --- | --- |
| Plant height | 1 |  |  |  |  |  |
| Panicle length | 0.896** | 1 |  |  |  |  |
| Tiller | 0.708* | 0.711 | 1 |  |  |  |
| Thousand grain weight | 0.243 | 0.178 | 0.087 | 1 |  |  |
| Grain yield | 0.861*** | 0.816** | 0.729* | 0.213 | 1 |  |
| Straw yield | 0.565* | 0.530* | 0.656* | 0.202 | 0.630* | 1 |

****P* < 0.001, ***P* < 0.01, **P* < 0.05, NS = not significant.

**S11 Table.** Correlation analysis of wheat yield and yield attributing traits at Parwanipur, Bara, Nepal.

|  | Plant height | Panicle length | Tiller | Thousand grain weight | Grain yield | Straw yield |
| --- | --- | --- | --- | --- | --- | --- |
| Plant height | 1 |  |  |  |  |  |
| Panicle length | 0.870** | 1 |  |  |  |  |
| Tiller | 0.304 | 0.321 | 1 |  |  |  |
| Thousand grain weight | 0.324 | 0.179 | 0.044 | 1 |  |  |
| Grain yield | 0.797** | 0.706* | 0.338 | 0.427 | 1 |  |
| Straw yield | 0.855*** | 0.792* | 0.483 | 0.372 | 0.909*** | 1 |

****P* < 0.001, ***P* < 0.01, **P* < 0.05, NS = not significant.
