# Supplementary material for: Potassium nutrient response in the rice-wheat cropping system in different agro-ecozones of Nepal
Source: PLoS One. 2021 Mar 18;16(3):e0248837. doi: 10.1371/journal.pone.0248837 (PMC7971854; doi:10.1371/journal.pone.0248837)
Supplement: Attachment — Submitted filename: Plagiarism report_Nepal.pdf [file pone.0248837.s003.pdf]

Plagiarism Checker X Originality Report

Similarity Found: 11%

Date: Tuesday, June 16, 2020
Statistics: 544 words Plagiarized / 4911 Total words
Remarks: Low Plagiarism Detected - Your Document needs Optional Improvement.

Revised potassium nutrition in rice-wheat cropping system of Nepal Potassium rate revision in rice-wheat cropping system 28 Abstract 29 Most of the soils of Nepal had a higher potassium (K) level in the past and only a minimum 30 rate of K fertilizer at 15 kg K2O ha-1 was recommended (RDKF) in rice-wheat cropping 31 system (RWCS) since 1976.

Those crops started showing K deficiency symptoms in recent 32 decades, which could be due to a large portion of soils with depleted K level or insufficient 33 input of K fertilizer for crop production. This study explored a limitation of K nutrition in 34 crops by establishing field trials at three agro-ecozones (high-Hills, inner-Terai, and Terai) 35 in Nepal.

Seven rates of K fertilizer as 0, 15, 30, 45, 60, 75, and 90 kg K2O ha-1 were 36 replicated four times in a randomized complete block design, where crop yields and yield 37 attributing parameters of RWCS were monitored. Results revealed that an increase in K 38 rates from 45 to 75 kg K2O ha-1 in inner-Terai and Terai and 45 to 60 kg ha-1 in high-Hills 39 conditions produced significantly higher grain yields compared to the RDKF.

A maximum 40 rate of K fertilizer should not exceed 68 kg K2O ha-1 for rice in all agro-ecozones, 73 kg K2O 41 ha-1 for wheat in inner-Terai and 60 kg K2O ha-1 for wheat in high-Hills and Terai as an 42 economically optimum rate of K fertilizer for crop production. Compared to the current 43 RDKF, a new rate of K fertilizer should be considered as at least four-times high for wheat 44 and three-times high for rice production.

Further study should be explored, including 45 different crop varieties (hybrids) and soil types for the agro-ecozones. 46 Keywords: Fertilizer recommendation; Hills; K fertility; Nepal; Nutrient response; Soil K; 47 Terai 48 49 50 51 52 53 1 Introduction 54 The rice (Oryza sativa L.) - wheat (Triticum aestivum L.) cropping system (RWCS) is the most 55 important cropping system in the sub-tropical zone of South and South-East Asia [1].

It 56 covers an estimated 13.6 million ha in Indo-Gangetic Plains (IGP) of India, Bangladesh, Nepal 57 and Pakistan [2]. It provides a livelihood for hundreds of millions of population that 58 contributes to regional food and nutritional security [3-4]. Rice - wheat crop rotation is also 59 the most common rotation system in Nepal [5-6].

In this system, rice is usually cultivated 60 during the warm monsoon season, while wheat is subsequently grown in the cold, humid 61 season. 62 Soils like Ustochrepts, Dystochrepts, Haplumbrepts are commonly found Inceptisols in 63 Nepal, which are dominant with illite, mica, chlorite, and kaolinite clay minerals [7-9]. 64 Inceptisols are fragile, weakly developed, and susceptible to management [10].

The 65 Inceptisols of Nepal were rich in K due to the presence of mica (a hydrous potassium 66 aluminium silicate mineral) and interlayer fixed K in 2:1 clay minerals, which contribute 67 most to the passive K pool but easy mineralization of mica also contribute in the active K 68 pool [11-12]. 69 In 1976, a soil test based fertilizer recommendation was made in Nepal by correlating crop 70 response data with soil test value deeming the importance of economic return per unit 71 fertilizer use [13]. Due to K rich soil, a low rate of K, i.e., 15 kg K2O ha-1 was recommended 72 for rice and wheat to maintain yield.

During these 43 years, no update on soil fertilizer rate 73 was made and farmers use minimal K fertilizer in the field except the input from organic 74 sources, predominantly farmyard manure [14-15]. Besides N as one of the major nutrients 75 for crop production, K plays a significant role in metabolic reaction in plants by activating a 76 multitude of enzymes and also has a positive effect on plant water stability and deficiency 77 causes reducing sugar accumulation and decreasing the organic acid [16].

78 Several studies reported a substantial change in K present in mineral parent material K and 79 fertilizer management in cropland [17-18] occurred in decennial time-scale. Subsequently, 80 the K consumption rate of Nepal in 2009 was 0.45 kg K2O ha-1 which is slightly increased in 81 2016 to 1.5 kg K2O ha-1 which is still far less than the RKDF of 15 kg K2O ha-1 [19].

Due to the 82 mineral dissociation and less K balance in soil, Nepal is now experiencing

a deficiency of K in the field crops. For instance, the yield of the RWCS is decreasing mainly due to a decline in soil fertility in recent decades [20-21]. Yields of the RWCS are also declining due to the incidence of diseases such as rusts, leaf blight, spot blotch [22] and temperatures in the region during grain filling period [23]. Many reports indicated that there is a decreasing trend of soil fertility, including K concentration in soils of Nepal [24-25].

A recent soil test report from soil testing mobile van program in 9 (out of 75) districts in Nepal revealed that around 33% of total sample tested (n= 1479) had soil K at 10-30 kg K2O ha-1 or even less [26]. Farmers use farmyard manure (FYM), mineralized K from FYM is primarily the K input in the soil. The current FYM application rate among Nepalese farmers is about 2.5-3 t ha-1 annually.

But, the quality of manure is inferior [27] that resulted in a very high FYM recommendation rate (20-30 t ha-1), which is far beyond farmers' achievability [28-29]. So, mineral K sources are necessary to replenish the mined K from the cropping soils. A proper nutrient management plan is necessary to sustain yield in the long run with a revision of possibly outdated K fertilizer rate.

Improved productivity of RWCS in Nepal with a revised rate of K fertilizer is also a major concern to keep up with population growth in Nepal, which is predicted to be 36 million by mid-2050 [30]. Previous K recommendations do not consider the environmental K response factor. Here, we select three agro-ecozones to address this shortcoming. Increasing K levels in the K deficient soils may increase crop productivity in RWCS.

So, we aimed to study the yield response and nutrient response of the additional K fertilizer in rice and wheat in three predominant RWCS agro-ecozones (inner-Terai, Terai, and high-Hills) of Nepal. 2 Materials and Methods 2.1 Study Area and Climate Nepal is divided into five agro-ecozones (Terai, inner-Terai, mid-Hills, high-Hills, and high- Himalayas) elevation ranges from 80 masl in Terai to 8,848 masl in high-Himalayas. Cultivation is done up to the 4,800 masl high-Hills [31].

The climate at all agro-ecozones is wet summer and dry winter subtropical. Average annual rainfall ranges from 1000-1800 mm and more than 80% of annual rainfall distributed from June to September, called monsoon period and the winter (November to February), is generally dry with scanty rainfall [32].

Three sites of the Nepal Agricultural Research Council (NARC) were selected in different agro-ecozones of Nepal. The first research site was established in

2009-2010 at National 115 Maize Research Program, Rampur, Chitwan which represents the inner-terai agro-ecozone 116 situated at the coordinates 27° 39' N, 84° 20' E and an elevation of 187 masl.

The second 117 research site was established in 2011-2012 at Hill Research Station, Kabre, Dolakha 118 representing high-Hill agro-ecozone with the coordinates 27° 38' N, 86° 9' E and an elevation 119 of 1820 masl. The third research site was established in 2012-2014 at Regional Agriculture 120 Research Station, Parwanipur, Bara represents the Terai agro-ecozones with the 121 coordinates 27° 4' N, 84° 55' E and an elevation of 96 masl (Figure 1). 122 [Figure 1] 123 Figure 1. Map showing the study area locations of Nepal.

124 During the research period, Kabre site received 2370 mm annual precipitation with 125 maximum rainfall (790 mm) in August and minimum (0 mm) rainfall from December to 126 February. The annual temperature ranges from 26? C (May and June) to 3? C (January). The 127 average maximum and minimum temperatures were 22? C and 12? C, respectively for the 128 region.

Similarly, Rampur site received 2290 mm annual precipitation with maximum 129 rainfall (697 mm) in August and minimum rainfall (0 mm) in November and January. The 130 annual temperature ranges from 37? C (April) to 10? C (December and January). The average 131 maximum and minimum temperatures were 31? C and 21? C, respectively. And, Parwanipur 132 site received 1423 mm annual precipitation with maximum rainfall (311 mm) in August and 133 minimum rainfall (0 mm) in November to January, and April. The annual temperature ranges 134 from 37.5? C (April) to 10? C (January). The average maximum and minimum temperatures 135 were 22? C and 12? C, respectively.

136 2.2 Soil 137 A baseline soil sampling from the top 20 cm depth was collected and analyzed for pH, OM, 138 total N, available P, and available K concentrations. The soil of Parwanipur (Terai) was 139 medium fertile silty clay soil with low in organic matter (OM, 1.1%), medium in total N 140 (0.11%), medium in available P (48 kg P2O5 ha-1), medium available K (165 kg K2O ha-1) and 141 slightly acidic pH (6.2).

Similarly, the soils of Kabre site (high-Hills) was low fertile sandy loam 142 soil with low in OM (1.3%), low in total N (0.07%), high in available P (107.3 kg P2O5 ha-1) 143 and medium in available K (121 kg K2O ha-1) and acidic soil pH (4.2). And the soils of Rampur 144 (inner-Terai) was medium fertile sandy loam soil with low in OM (2.2%), medium in N 145 (0.14%), high in available P (118 kg P2O5 ha-1), low in available K (61 kg K2O ha-1) and slightly 146 acidic soil pH (5.8).

Soil samples from the top 20 cm were again collected from experimental 147 plots in 2012 (Kabre site), 2010 (Rampur site) and 2013 (Parwanipur site) to observe 148 changes in pH, OM, total N, available P, and available K concentrations before planting of 149 the main crop. 150 2.3 Experimental Design and Treatment 151 At all three sites, the experimental design was layout in Randomized Complete Block Design 152 with four replications.

Treatment included seven rates of K fertilizer as 0, 15, 30, 45, 60, 75 153 and 90 kg K2O ha-1. The N and P fertilizers were applied at the rate of 100 kg N ha-1 and 30 154 kg P2O5 ha-1 in each plot. Half rate of N, full rate of P, and full rate of K was applied as basal 155 at the time of transplanting and the remaining half N was applied at the tillering stage.

156 Urea, triple superphosphate, and muriate of potash were sources of N, P, and K which 157 contains 46% N, 46% P2O5, and 60% K2O, respectively. All these fertilizers were sourced from 158 a local agro-vet shop. Similarly, rice and wheat seeds were sourced from the Botany Division 159 of NARC, which were the recommended varieties for the regions.

WK 1204 variety of wheat 160 and Ram Dhan variety of rice was selected for high-Hill region and Gautam variety of wheat 161 and Hardinath variety of rice was selected for inner-Terai and Terai regions. Seeds of wheat 162 were directly sown in the field, however, 21 days old seedlings of rice were transplanted in 163 the puddled field in a row-row spacing of 20 cm and hill to hill spacing of 20 cm. 164 2.4 Statistical Analysis 165 Yield and yield parameters of rice and wheat were collected during the growing seasons.

166 Length parameters (plant height and panicle length) were measured by using scale and 167 weight parameters (grain yield, straw yield, biomass, and thousand-grain weight) were 168 measured. Tiller number was recorded with manual counting. Relative yield (RY) was 169 calculated by subtracting control yield from treatment yield (delta yield) and divided by 170 170 171 171 control yield and expressed in percentage.

???? (%) = ?????????? ?????????? ?? 100 ?????????????? ?????????? 172 The maximum physical K rate was calculated by equating the first-order derivative of yield 173 response function with zero and the economic optimum K rate was calculated by the first- 174 174 175 175 order derivative of yield response function with price ratio [33].

???? For maximum physical K rate, ???? 176 ???? ???? For economic optimum K rate, ???? = ???? 177 Where, ?y = yield response function, ?x = derivative for x, Py = unit price of

fertilizer, 178 Px = unit price of grain yield. 179 Collected data were compiled and cleaned in MS Excel program. The assumption of analysis 180 of variance (ANOVA) was tested and means were separated at a 5% level of significance 181 using Tukey's test using R-studio.

Time (in years) and location were not combined to analyze 182 as fixed variables, and they were treated individually. A correlation test was carried out 183 between different parameters at a 95% level of significance. 184 3 Results 185 3.1 Soil chemical properties 186 In a year, changes in soil chemical properties due to addition of different rates of K fertilizer 187 were measured after harvest of the first year's crop (in different years: Kabre in 2012, 188 Rampur in 2010 and Parwanipur in 2013) at different sites and their values are presented in 189 Supplemental Table S1. There were no significant changes in pH, OM, N and P (except in 190 Rampur site) in addition to K at different rates.

Soil K concentration increased with addition 191 of K rates in Kabre site compared to control. Only K rates at 60 kg K2O ha-1 or higher 192 increased soil K concentration compared to control in Rampur site (Supplemental Table S1). 193 3.2 Yield Response 194 3.2.1

Inner Terai (Rampur) 195 Thousand-grain weight, grain yield and straw yield of rice and wheat increased with the 196 addition of K fertilizer in the inner-Terai condition (Table 1). Results suggested that there 197 was more demand for K fertilizer to obtain a significantly higher yield of rice than RDKF, i.e., 198 15 kg K2O ha-1 recommended rate of K fertilizer.

Potassium fertilizer rate at least 45 kg K2O 199 ha-1 or higher produced significantly greater yields, with a trend of r2=0.69, P=0.122 in 2009, 200 r2=0.55, P=0.26 in 2010 and r2=0.94, P=0.006 in 2011. Similarly, a significantly higher rice 201 straw yield was obtained from 75 kg K2O ha-1 than RDKF in the first and third years. However, 202 rice yield was declining up to 30-33% in the second year (Table 1). 203 Table 1.

Analysis of variance (ANOVA) results with means for thousand-grain weight, grain yield and straw yield of rice as affected by different rates of potassium 204 fertilizer in rice-wheat cropping system at NMRP, Rampur, Chitwan, Nepal in three consecutive years. Thousand-grain weight, g Grain yield, kg ha-1 Straw yield, kg ha-1 205 †Treatments included 0 (or control), 15, 30, 45, 60, 75, and 90 kg K2O ha-1.

206 ‡Means in a column followed by same lowercase letter are not significantly different. 207 *P < 0.05,* *P < 0.01, ***P < 0.001 and NS = not significant. 208 Thousand-grain weight (TGW) of rice was significantly different than RDKF in the first and 209 third years

from 75 and 60 kg K2O ha-1, but no significant difference was observed in second 210 year (Table 1).

Plant height was affected by K rate 75 kg K2O ha-1 and significantly higher 211 than RDKF, but panicle length and tillers number did not differ significantly with RDKF 212 (Supplemental Table 2). All these yields attributing traits were positively correlated with 213 yield, but a strong positive correlation (r2 = 0.87, p = 0.000) was reported with plant height 214 (Supplemental Table 5).

215 Wheat grain and straw yield significantly differ from RDKF with an additional level of K 216 (Table 2). The significant yield of rice recorded from 75 to 45 kg K2O ha-1 and wheat from 90 217 to 60 kg K2O ha-1 in the first and second year, respectively. And subsequent decline of 38% 218 in grain yield and 20% in straw yield was observed over years (Table 2). 219 Table 2.

ANOVA results with means for thousand-grain weight, grain yield and straw yield of wheat as affected by different rates of potassium fertilizer in rice-wheat 220 cropping system at NMRP, Rampur, Chitwan, Nepal in three consecutive years. Thousand-grain weight, g Grain yield, kg ha-1 Straw yield, kg ha-1 221 †Treatments included 0 (or control), 15, 30, 45, 60, 75, and 90 kg K2O ha-1.

222 ‡Means in a column followed by same lowercase letter are not significantly different. 223 ***P < 0.001 and NS = not significant. 224 224 225 225 226 226 227 Thousand-grain yield was significantly different at 60 kg K2O ha-1 than RDKF. Plant height 228 was significantly different than RDKF with the highest plant height reported from 90 kg K2O 229 ha-1 in the first and 75 kg K2O ha-1 in the second years (Table 2).

There was no significant 230 difference in panicle length and tiller number with RDKF and no significant difference in 231 tillers number with control on the second and third year (Supplemental Table S4). Highest 232 positive correlation was observed between wheat grain yield and plant height (r2 = 0.815, p 233 = 0.000) and panicle length (r2 = 0.743, p = 0.000) (Supplementary Table S5). 234 3.2.2

High Hills (Kabre) 235 Additional K rate had no significant response over RDKF in straw yield and grain yield of 236 wheat in both year and rice in the first year (Table 3). Rice grain and straw yield were 237 significantly different in the second year of potassium application with 30 kg K2O ha-1. Over 238 the years, grain yield of rice increased by 10%, but straw yield declined by 22%. 239 Table 3.

ANOVA results with means for thousand-grain weight, grain yield and straw yield of rice

and wheat as affected by different rates of potassium fertilizer in rice- 240 wheat cropping system at HRS, Kabre, Dolakha, Nepal in two consecutive years. Rice Wheat (2011) Thousand-grain weight, g Grain yield, kg ha-1 Straw yield, kg ha-1 Thousand-grain weight Grain yield, Straw yield, 241 †Treatments included 0 (or control), 15, 30, 45, 60, 75, and 90 kg K2O ha-1. 242 ‡Means in a column followed by same lowercase letter are not significantly different. 243 ** P < 0.01, ***P < 0.001 and NS = not significant 244 Thousand grain weight of the rice not significantly different in the first and second year 245 (Table 3).

Plant height of rice significantly differed at 45 kg K2O ha-1 application in the second 246 year, but tiller number, panicle length of rice and plant height and tiller numbers of wheat 247 were not significantly different with RDKF (Supplemental Table S6). Panicle length of wheat 248 was significantly different at 60 kg K2O ha-1 than RDKF.

Significant correlation of grain yield 249 was observed with plant height of rice (r2 = 0.805, p = 0.000), plant height of wheat (r2 = 250 0.682, p = 0.000), and tillers number (r2 = 0.752, p = 0.000) (Supplemental Tables S7 and S8). 251 3.2.3 Terai (Parwanipur) 252 Grain yield and straw yield significantly differed from control but not with RDKF in Rice 253 (Table 4).

But, a significant response of additional K fertilizer was reported from 60 Kg K2O 254 ha-1 in wheat. An incremental yield of rice was achieved up to 60 kg K2O ha-1 but declined 255 afterward. Over the years, yield increased up to 30% and incremental highest yield over 256 control treatment was reported up to 80%.

The addition of K did not increase the thousand- 257 grain of rice in both years. A 33% increase in wheat grain yield was reported from 60 kg K2O 258 ha-1 compared to the RDKF. Wheat straw yield was similar (3.7 t ha-1) at 60 kg K2O ha-1 in 259 both years. 260 Table 4.

ANOVA results with means for thousand-grain weight, grain yield and straw yield of rice and wheat as affected by different rates of potassium fertilizer in rice- 261 wheat cropping system at RARS, Parwanipur, Bara, Nepal. Rice Wheat Thousand-grain Thousand-grain weight, g Grain yield, kg ha-1 Straw yield, kg ha-1 weight, g Grain yield, kg ha-1 Straw yield, kg ha-1 Treatment† 2012 2014 2012 2014 2012 2014 2013 2014 2013 2014 2013 2014 0 19.9 22.6 1507.0b‡ 1551.0c 401.0b 3953.0b 41.7b 45.5b 761.0b 1029.0d 1196.0d 1282.0d 15 22.1 22.1 2287.0a 2993.0ab 6823.0a 6691.0a 43.3b 46.3b 2474.0a 2452.0c 3241.0c 2746.0c 30 21.1 21.6 2423.0a 2997.0ab 6351.0a 7492.0a 45.9a 47.2ab 2614.0a 2777.0bc 3472.0bc 3170.0b 45 21.5 23.2 2095.0a 2344.0b 6702.0a 6018.0a 46.3a 44.8b 2475.0a 2829.0abc 3412.0bc 3211.0b 60 22.5 23.4 2623.0a 3333.0a

6707.0a 7104.0a 47.7a 46.9b 2767.0a 3310.0a 3770.0a 3776.0a 75 22.9 23.1 2092.0a 2772.0ab 6827.0a 6778.0a 47.4a 47.3ab 2633.0a 3055.0ab 3655.0ab 3627.0a 90 23.3 22.9 2159.0a 3244.0a 6917.0a 7160.0a 47.3a 50.3a 2556.0a 3038.0ab 3865.0a 3431.0ab Significance NS NS ** *** *** *** *** * *** *** *** *** 262 †Treatments included 0 (or control), 15, 30, 45, 60, 75, and 90 kg K_2O ha^{-1}.

263 ‡Means in a column followed by same lowercase letter are not significantly different. 264 *$P < 0.05$,* *$P < 0.01$, ***$P < 0.001$ and NS = not significant. 265 Thousand grain of wheat was obtained significantly from 30 kg K_2O ha^{-1} in the first year and 266 90 Kg K_2O ha^{-1} in the second year (Table 4). In both crops except tillers number other yield 267 attributes traits (plant height and panicle length) significantly differ from control but similar 268 with RDKF.

Significantly the highest tillers number of rice (260 m^{-2}) and wheat (312 m^{-2}) was 269 obtained from 90 Kg K_2O ha^{-1} (Supplemental Table S9). Strong positive significant 270 correlation exists between yield and yield attributing traits (plant height and panicle length) 271 of rice ($r^2 = 0.82$, $p = 0.000$) and wheat ($r^2 = 0.76$, $p = 0.000$) but a weak non-significant 272 correlation ($r^2 = 0.32$, $p = 0.87$) with thousand grain weight (Supplemental Tables S10 and 273 S11).

274 3.3 Nutrient Response 275 3.3.1 Physical Maximum and Economic Optimum Rate 276 Physical maximum and economic optimum rates of K for rice and wheat at all agro ecozones 277 were near values (Table 5). Physical maximum rate of the K for maximum production was 278 required highest for the wheat in inner-Terai which is 4.8

times the recommended rate and 279 the least for the rice in Terai which is 3.4 times the recommended rate. Similarly, the K 280 requirement for the wheat should be increased by 4.7 times in inner-Terai and 3.9 times in 281 high-Hills and Terai. 282 282 283 Table 5. Physical maximum rate and economic optimum rate of potassium fertilizer recommended 284 for the rice-wheat cropping system in different agro-ecozones of Nepal.

Agro-ecozone Crop Nutrient response curve function $y = -0.4627x^2 + 58.401x +$ Physical maximum Economic optimum rate rate kg ha^{-1} high-Hills (Dolakha) Rice Wheat inner-Terai Rice (Chitwan) Wheat Terai (Bara) Rice Wheat 2397.1 $y = -0.5017x^2 + 72.826x + 634.0$ $y = -0.2535x^2 + 30.839x + 1812.2$ $y = -0.4946x^2 + 60.738x + 1197.4$ $y = -0.3245x^2 + 41.085x + 2121.3$ $y = -0.3983x^2 + 47.665x + 1255.3$

285 285 286 3.3.2 Expected Yield and Relative Yield over Residual Fertility Yield 287 Additional K rate increased the relative yield of rice and wheat over soil residual fertility

288 with incremental expected yield (Figure 2). The residual fertility rice yield is around 2 t ha-1 289 at all agro-ecozones.

The addition of K rate was expected to increase the yield of rice 290 maximally by 1.8, 0.9, and 1.3 t ha-1 in inner-Terai, Terai, and high-Hills, respectively. 291 Similarly, the residual fertility yield of wheat was 0.6 t ha-1 in inner-Terai and around 1 t ha- 292 1 in Terai and high-Hills. The addition of K rate was expected to increase the yield of wheat 293 maximally by 2.6, 1.8, 1.4 t ha-1 in inner-Terai, Terai, and high-Hills, respectively.

The 294 response of K was high in inner-Terai due to the low residual potassium level. 295 295 296 [Figure 2] 297 Figure 2. Aggregated data analysis to compare the different levels of potassium application on expected yield 298 (represents by bar graph) and relative yield (represents by green line graph) over residual fertility yield 299 (represent by a straight blue line) in rice (Figure 2A, 2C, 2E) and wheat (Figure 2B, 2D, 2F) in inner-Terai (2A, 300 2B), Terai (2C, 2D), and high-Hills (2E, 2F).

301 4 Discussion 302 Increased yields of rice and wheat at inner Terai and Terai regions of Nepal with K rate at 303 not less than 60 kg K2O ha-1 evident that these regions have low availability of nutrients due 304 to sandy soil, high rainfall intensity, and frequent leaching loss of K [34]. Other findings also 305 reported low availability of nutrients in those regions [5, 35-36].

The soils of these regions 306 are generally characterized as fertile soil, which is made up of recent alluvial deposits mostly 307 fine sand and silt with light to medium texture, and even Terai region of Nepal is considered 308 as the basket of grain [37-38]. Crop cultivation in this region started after clearing the forest 309 in 1927 [39].

A robust agricultural production was witnessed with an alluvial deposit, 310 medium-textured, forest soils. But in later decades, low organic input, nutrient removal due 311 to crop harvesting, heavy tillage work, continuous erosion, and poor crop and land 312 management resulted in low nutrient reserve [40-41]. Hence, K, an active part of the 313 nutrient cycle, is also impacted by low nutrient reserve.

314 In high-Hills, increased crop yields were recorded with an additional K rate in the second 315 year from 30 kg K2O ha-1 not exceeding 70 kg K2O ha-1. Soil in the region is evident with low 316 in OM content, high erosion rate, and sandy loam texture with acidic soil pH [42-43]. 317 Additionally, mid-and-high-Hills are characterized by erosion rate of 32-38 t ha-1 yr-1 [44].

318 Carson [45] reported a loss of 1 mm topsoil had been estimated to cause loss of

10-kg 319 nitrogen ha-1, 7-kg P ha-1 and 15 kg hectare ha-1. Around 3000 kg K ha-1 has been removed 320 from the soil with the productivity of rice and wheat 7 and 5 t ha-1, respectively [46]. In 321 contrast, rice-growing farmers in the mid-hill regions have benefited from the accumulation 322 of eroded sediments (for example, mica), a source of exchangeable K [47-49]. 323 Comparing the K fertilizer consumption under RWCS in the IGP regions, Nepal uses less 324 amount of potassium fertilizer in rice (0.8

kg ha-1) and wheat (1.7 kg ha-1) compared to China 325 in rice (33.2 kg ha-1) and wheat (26.6 kg ha-1). Other countries, such as India uses 7.8 kg ha- 326 1 in rice and 3.2 kg ha-1 in wheat, whereas Bangladesh uses 8.3 kg ha-1 in rice and 6.6 kg ha- 327 1 in wheat [2]. A long term rice-wheat fertility experiment conducted in Bhairahawa, Nepal 328 showed that annual K balance was negative and K showed a large response in rice [5].

The 329 increase in yield of rice and wheat was evident in all three agro-eco zones of Nepal. 330 Nutrient response functions showed the physical and economic limits of K rate were more 331 or less equal at all locations. The K requirement of the rice should be increased by 4.4, 3.8, 332 3.3 times RDKF in high-Hills, inner-Terai, and Terai, respectively.

In the current study, the 333 economic optima of the K ranged from 50-67 kg K2O ha-1 in rice and 58-71 kg K2O ha-1 in 334 wheat in different locations. The availability of K in soil is proportional to the additional K 335 fertilizer [50], but a higher rate of K addition results in fixation rather than availability [48]. 336 Potassium being a 'luxury consumption' nutrient, sometimes additional K may result in loss 337 [51].

Also, K is a mobile element additional K results in leaching under flooded conditions in 338 case of rice cultivation [49]. So, the current study was unable to attain a more economic 339 yield in 90 kg K2O ha-1. Hence it is not worthy of applying more than 70 kg K2O ha-1 starting 340 from 50 kg K2O ha-1 depending upon crop type and agro-eco zone.

341 At all locations, an additional K rate increased relative yield between 40-50 and 60-80% in 342 rice and wheat. The rice yield was estimated to increase up to 3 to 4.5 t ha-1 and wheat yield 343 was estimated to increase 2.5-3 t ha-1 from 60 kg K2O ha-1. This different K response in rice 344 and wheat is witnessed due to the K limitation in the soil and the availability of K after K 345 fertilizer application.

As the soils of the studied region were high in mica content. In soils 346 containing high mica, even 1-2 % of total K is enough. However, continual crop removal of 347 K and

restricted K application in soil for a long time resulted in the weathering of mica to 348 biotite or vermiculite, which is an avenue for K limitation [52]. Similarly, the exchange of K 349 between available and fixed pools is possible.

More than 50% of total K availability in RWCS 350 is obtained from fixed K pools that may further deplete the K reserve from the soil [36]. So, 351 it is imperative to apply K fertilizer to maintain K levels and soil fertility in the cropland. 352 The current study reported that there were no differences in soil total N after a year of 353 addition of K fertilizer, suggesting no adverse effects on total N due to treatment 354 application.

These days, Nepalese farmers are aware of the importance of chemical 355 fertilizers in crop production and the application of chemical fertilizers is gradually 356 increasing. Most of the farmers use only N related fertilizers, which may reach the potential 357 yields. However, it can increase the cost of cultivation in short term and reduction in soil 358 quality and productivity in long term.

A continuous and increased application N fertilizer is 359 not enough to replenish lost plant nutrients and maintain soil productivity [25, 53]. So, 360 balanced fertilization is necessary to increase the productivity of RCWS. 361 To summarize, the current study recommends revising the RDKF (15 kg K2O ha-1) to 60-70 362 kg K2O ha-1 in different agro-eco zones.

The use of a single variety of rice and wheat, which 363 was suitable to the soils of research sites, could be a potential pitfall of this study. The K 364 rate might differ for hybrid cultivars of rice and wheat and different soil types. So, a series 365 of experiments are suggested with different varieties of crop in different soil types in 366 different crop rotations.

367 5 Conclusion 368 Improvements in crop yield and soil nutrient response with addition of K fertilizer in RWCS 369 at three agro-ecozones of Nepal suggests that the current recommendation rate of K 370 fertilizer should be increased about 3-4 times for rice and 4-4.5 times for wheat to achieve 371 optimum economic production. These recommendations are made for the improved 372 varieties with the yield potential 2-4 t ha-1 of rice and wheat, which may vary for other high 373 yielding varieties.

Fertilizer recommendation is a dynamic and continuous process that 374 largely depends on soil type, crop response, inherent fertility, grain to fertilizer price ratio, 375 and environment. Recommendations should be revised regularly considering these factors 376 over time. Therefore, K, an integral crop nutrient component contributing to soil fertility 377 and optimum crop production, should be applied in the recommended

rate in a balanced 378 way appraising the available K sources.

INTERNET SOURCES:

<1% - https://www.ipipotash.org/publications/eifc-216
<1% - https://projects.sare.org/project-reports/os09-048/
<1% - https://www.frontiersin.org/articles/10.3389/fnut.2019.00081/full
<1% - https://mafiadoc.com/soil-fertility-and-plant-nutrient-management_5c1ef5ad097c4772298b45b1.html
<1% - https://www.researchgate.net/publication/283127962_Response_of_maize_yield_quality_and_nitrogen_use_efficiency_indices_to_different_rates_and_application_timings
<1% - https://www.researchgate.net/publication/251449054_Rice-Wheat_Cropping_Systems
<1% - https://www.researchgate.net/publication/249995538_Reprint_of_Optimizing_intensive_cereal-based_cropping_systems_addressing_current_and_future_drivers_of_agricultural_change_in_the_Northwestern_Indo-Gangetic_Plains_of_India
<1% - http://www.fao.org/3/a-i5555e.pdf
<1% - https://www.sciencedirect.com/science/article/pii/S0959652620303668
<1% - https://www.researchgate.net/publication/230512903_Potassium_replenishment_capacity_of_illitic_soils_at_their_minimal_exchangeable_K_in_relation_to_clay_mineralogy
<1% - https://en.wikipedia.org/wiki/Natural_mineral
<1% - https://www.sciencedirect.com/science/article/pii/S0167198717300533
<1% - https://link.springer.com/10.1007/s10533-012-9802-4
<1% - https://www.researchgate.net/publication/277309038_Role_of_iron_in_plant_growth_and_metabolism
<1% - https://link.springer.com/article/10.1007/s11104-019-04229-0
<1% - https://www.researchgate.net/publication/328580200_Assessment_of_Soil_Fertility_under_Different_Land-Use_Systems_in_Dhading_District_of_Nepal
<1% - https://journals.plos.org/plosone/article?id=10.1371/journal.pone.0206350
<1% - https://www.researchgate.net/publication/287106173_Sustainability_issues_on_rice-wheat_cropping_system

<1% - https://www.sciencedirect.com/science/article/pii/S2214581814000329
<1% -
https://www.researchgate.net/publication/272354425_Impacts_Assessment_and_Climate
_Change_Adaptation_Strategies_in_Makawanpur_District_Nepal
<1% -
https://shodhganga.inflibnet.ac.in/bitstream/10603/90811/11/11_chapter%203.pdf
<1% -
https://www.researchgate.net/publication/326293119_Evaluation_of_Hybrid_and_OPV_M
aize_Varieties_for_Grain_Yield_and_Agronomic_Attributes_under_Farmer's_Field_Conditio
n_at_Dukuchhap
<1% -
https://www.researchgate.net/publication/338540601_Evaluation_of_rice_genotypes_for_
its_response_to_added_fertility_levels_and_induced_drought_tolerance_during_reproduct
ive_phase
<1% - https://www.climatestotravel.com/climate/indonesia/sumba
<1% - https://en.wikipedia.org/wiki/Temperature_in_Canada
<1% -
https://mafiadoc.com/abstracts-indian-society-of-soil-science_5a5a0d4f1723dd5880daa
1dc.html
<1% - https://www.ncbi.nlm.nih.gov/pmc/articles/PMC6804989/
<1% -
https://www.researchgate.net/publication/265469230_ROCK_PHOSPHATE_ITS_AVAILABI
LITY_AND_SOLUBILIZATION_IN_THE_SOIL_-_A_REVIEW
<1% - https://www.sciencedirect.com/science/article/pii/S0167880905003634
<1% - https://www.sciencedirect.com/science/article/pii/S0378429005002765
<1% -
https://www.researchgate.net/publication/249077637_Potassium_Phosphorus_Sulfur_an
d_Boron_Fertilization_Effects_on_Soybean_Isoflavone_Content_and_Other_Seed_Charact
eristics
<1% -
https://www.researchgate.net/publication/328476598_Estimating_Soil_Nutrient_Supply_
Capacity_for_Rice_Oryza_sativa_L_Production
<1% - http://www.pjar.org.pk/Issues/Vol25_2012No_4/Vol25No_4Page266.pdf
<1% -
https://www.researchgate.net/publication/298869390_Response_of_Vegetables_to_NPK_
Briquette_Deep_Placement
<1% -
https://www.researchgate.net/publication/330838895_Simulation_of_growth_and_yield_
of_rice_and_wheat_varieties_under_varied_agronomic_management_and_changing_clima
tic_scenario_under_subtropical_condition_-_Copy

<1% -
https://www.researchgate.net/publication/234064947_Efficiency_of_Phosphogypsum_and_Mined_Gypsumin_Reclamation_and_Productivity_of_Rice-WheatCropping_System_in_Sodic_Soil
<1% - https://scialert.net/fulltext/?doi=ijar.2007.704.711
<1% - https://www.sciencedirect.com/science/article/pii/S0929139319310005
<1% - https://www.sciencedirect.com/science/article/pii/S096706371730136X
<1% -
https://www.researchgate.net/publication/306379153_Yield_response_of_wheat_to_nitrogen_and_potassium_fertilization
<1% -
https://www.researchgate.net/publication/229569590_Studies_on_Soil_Potassium_II_The_Immediate_QI_Relations_of_Labile_Potassium_in_the_Soil
<1% -
https://www.researchgate.net/publication/263036501_Effect_of_Integrated_Application_of_Bio-fertilizer_on_Grain_Yield_Yield_Components_and_Associated_Traits_of_Maize_Cultivars
<1% - http://pubs.sciepub.com/wjar/2/4/6/
<1% - https://www.sciencedirect.com/science/article/pii/S0168160518306342
<1% -
https://mafiadoc.com/advances-in-islamic-economics-and-finance_5a79d93c1723dd7a5b425049.html
<1% - https://www.ncbi.nlm.nih.gov/pmc/articles/PMC4964311/
<1% - http://www.infrc.or.jp/knf/PDF%20KNF%20Conf%20Data/C2-6-046.pdf
<1% - http://www.cropj.com/petroudi_5_5_2011_566_574.pdf
<1% - https://www.sciencedirect.com/science/article/pii/S1672630817300525
<1% - https://www.sciencedirect.com/science/article/pii/S0378429019303090
<1% - https://mafiadoc.com/es-i-jam-mu-2-0-1-6_59b4f12e1723ddd9c6442334.html
<1% - http://wssaabstracts.com/public/59/proceedings.html
<1% -
https://www.researchgate.net/publication/238087532_The_Effect_of_Straw_Stubble_and_Potassium_on_Grain_Yield_of_Rice_in_Rice-Rice_Cropping_Systems_in_the_Mid-Country_Wet_Zone_of_Sri_Lanka
<1% -
https://www.researchgate.net/publication/324953556_Evaluation_of_wheat_performance_under_different_sowing_techniques_in_agro_climatic_conditions_of_KPK_Pakistan
<1% - https://nepis.epa.gov/Exe/ZyPURL.cgi?Dockey=P1002Z4M.TXT
<1% - https://www.sciencedirect.com/science/article/pii/S0038071710004529
<1% - https://acsess.onlinelibrary.wiley.com/doi/full/10.2135/tppj2018.07.0005
<1% -

https://www.researchgate.net/publication/332963865_MORPHOLOGICAL_CHARACTERIZATION_OF_SWEET_SORGHUM_GENOTYPES_ACROSS_ENVIRONMENTS
<1% -
https://www.researchgate.net/publication/226583020_Estimation_of_dry_matter_and_starch_concentration_in_potatoes_by_determination_of_under-water_weight_and_near_infrared_spectroscopy
<1% -
https://www.thefreelibrary.com/EFFECT+OF+LONG+TERM+FERTILIZATION+ON+GRAIN+YIELD+AND+YIELD+COMPONENTS...-a0546690870
<1% - https://sites.google.com/site/soilgautamshrestha/
<1% -
http://www.aup.edu.pk/sj_pdf/EFFECT%20OF%20PHOSPHORUS%20ON%20THE%20YIELD%20AND%20YIELD%20COMPONENTS%20OF%20WHEAT%20VARIETY%20%93INQLAB-91%94%20UNDER%20RAINFED%20CONDITIONS.pdf
<1% - https://www.sciencedirect.com/science/article/pii/S0963996919307410
<1% -
https://www.researchgate.net/publication/328989156_Nickel_content_in_grain_of_winter_wheat_Triticum_aestivum_L_and_spring_barley_Hordeum_vulgare_L_cultivated_on_loessial_soil_depending_on_liming_and_mineral_fertilization
<1% -
https://www.researchgate.net/publication/324021438_The_Effects_of_Tillage_and_Straw_Incorporation_on_Soil_Organic_Carbon_Status_Rice_Crop_Productivity_and_Sustainability_in_the_Rice-Wheat_Cropping_System_of_Eastern_China
<1% -
https://www.scribd.com/document/369592107/Journal-of-Maize-Research-and-Development-Vol-2-No-1-2016
<1% - https://www.sciencedirect.com/science/article/pii/S0167198718303040
<1% -
https://slidelegend.com/rice-almanac-books-irri_5a48dfd01723ddcc66a9c36e.html
<1% -
https://www.researchgate.net/publication/226936520_Geological_and_geochemical_examination_of_arsenic_contamination_in_groundwater_in_the_Holocene_Terai_Basin_Nepal
<1% -
https://www.researchgate.net/publication/276341813_Harnessing_the_climate_mitigation_conservation_and_poverty_alleviation_potential_of_seagrasses_Prospects_for_developing_blue_carbon_initiatives_and_payment_for_ecosystem_service_programmes
<1% -
https://pdfs.semanticscholar.org/9d24/c93e72b5902f54457075c1350d5af7d7f366.pdf
<1% -
https://www.researchgate.net/publication/272073156_Rice_residue-_management_optio

ns_and_effects_on_soil_properties_and_crop_productivity
<1% - https://www.researchgate.net/publication/228941315_Managing_Seasonal_Soil_N-dynamics_in_Rice_Wheat_Rotation_Systems_of_Nepal
<1% - https://link.springer.com/article/10.1007%2Fs12571-015-0492-3
<1% - https://www.adb.org/sites/default/files/linked-documents/48218-003-ieeab.pdf
<1% - https://www.researchgate.net/publication/334597015_Effect_of_different_levels_of_nitrogen_and_potassium_on_the_growth_and_yield_of_okra
<1% - https://www.sciencedirect.com/science/article/pii/S0944501313001432
<1% - https://www.researchgate.net/publication/320550585_Effect_of_split_application_of_potassium_and_nitrogen_on_wheat_Triticum_aestivum_growth_and_yield_under_temperate_Kashmir
<1% - https://www.researchgate.net/publication/26255925_Phosphorus_A_Looming_Crisis
<1% - https://www.nature.com/articles/s41598-019-38697-2
<1% - https://www.agriculturejournal.org/volume2number1/tillage-system-crop-residues-and-nitrogen-to-improve-the-productivity-of-direct-seeded-rice-and-transplanted-rice/
<1% - https://www.researchgate.net/publication/275152771_Yield_trends_and_nutrient_budgeting_under_a_long-term_28_years_nutrient_management_in_rice-wheat_cropping_system_under_subtropical_climatic_condition
<1% - https://www.researchgate.net/publication/316276429_Participatory_potato_seed_production_a_breakthrough_for_food_security_and_income_generation_in_the_central_highlands_of_Ethiopia
