# Supplementary material for: Potassium nutrient response in the rice-wheat cropping system in different agro-ecozones of Nepal
Source: PLoS One. 2021 Mar 18;16(3):e0248837. doi: 10.1371/journal.pone.0248837 (PMC7971854; doi:10.1371/journal.pone.0248837)
Supplement: Attachment — Submitted filename: Plag Potassium_Nepal Revised.pdf [file pone.0248837.s007.pdf]

Plagiarism Checker X Originality Report

Similarity Found: 9%

Date: Friday, October 23, 2020
Statistics: 583 words Plagiarized / 6453 Total words
Remarks: Low Plagiarism Detected - Your Document needs Optional Improvement.

Abstract 29 Most of the soils of Nepal had a higher potassium (K, expressed as K2O) level in the past, 30 and since 1976 K fertilizer rate at 30 kg K2O ha-1 has been recommended in rice-wheat 31 cropping systems (RWCS). Those crops began showing K deficiency symptoms in recent 32 decades, which could be due to a large portion of soils with depleted K level or the 33 insufficient input of K fertilizer for crop production.

This study explored a limitation of K 34 nutrition in crops by establishing field trials from 2009-2014 at three agro-ecozones (inner- 35 Terai (2009-2010), high-Hills (2011-2012), and Terai (2012-2014)) in Nepal. Seven rates of 36 K fertilizer at 0, 15, 30, 45, 60, 75, and 90 kg K2O ha-1 were replicated four times in a 37 randomized complete block design, where crop yields and yield-attributing parameters of 38 RWCS were monitored.

Results revealed that an increase in K rates from 45 to 75 kg K2O 39 ha-1 under inner-Terai and Terai conditions and 45 to 60 kg ha-1 under high-Hills conditions 40 produced significantly higher mean response of grain yields compared to the 41 recommended K dose. However, the economic optimum rate of K fertilizer should not 42 exceed 68 kg K2O ha-1 for rice in all agro-ecozones, or 73 kg K2O ha-1 for wheat in inner- 43 Terai and 60 kg K2O ha-1 for wheat in high-Hills and Terai. Further study is needed that 44 incorporates different crop varieties (hybrids) and soil types for these agro-ecozones.

45 Keywords: Fertilizer recommendation; Hills; K fertility; Nepal; Nutrient response; Soil K; 46 Terai 47 1 Introduction 48 The rice (Oryza sativa L.)-wheat (Triticum aestivum L.) cropping system (RWCS) is the most 49 important cropping system in the subtropical zone of South and Southeast Asia [1]. It covers 50 an estimated 13.6

million ha in the Indo-Gangetic Plains (IGP) of India, Bangladesh, Nepal, 51 and Pakistan [2] and provides a livelihood for hundreds of millions of people that 52 contributes to regional food and nutritional security [3-4]. Rice-wheat crop rotation is also 3 the most common rotation system in Nepal [5-6]. In 53 this system, rice is usually cultivated 54 during the warm monsoon season (June to September), while wheat is subsequently grown 55 in the cold, humid season (November to February).

56 Soils such as Ustochrepts, Dystochrepts, and Haplumbrepts are common Inceptisols in 57 Nepal, which are dominant with illite, mica, chlorite, and kaolinite clay minerals [7-9]. 58 Inceptisols are fragile, weakly developed, and susceptible to management [10]. The 59 Inceptisols of Nepal were rich in K due to the presence of mica (a hydrous potassium 60 aluminum silicate mineral) and interlayer fixed K in 2:1 clay minerals that contribute most 61 to the passive K pool, though easy mineralization of mica also contributes to the active K 62 pool [11-12].

63 In 1976, a soil test-based fertilizer recommendation was made in Nepal by correlating crop 64 response data with soil test values deeming the importance of economic return per unit of 65 fertilizer use [13]. Due to the K rich soil, a low rate of K (30 kg K2O ha-1) was recommended 66 for rice and wheat to maintain yield. During the past 44 years, no update on soil fertilizer 67 rate has been made and farmers use minimal K fertilizer in the field apart from organic 68 sources, predominantly farmyard manure [14-15].

While N is one of the major nutrients for 69 crop production, K plays a significant role in metabolic reactions in plants by activating a 70 multitude of enzymes and creating a positive effect on plant water stability and deficiency 71 causes, reducing sugar accumulation and decreasing organic acid [16]. 72 Several studies reported a substantial change in K present in mineral parent material K and 73 fertilizer management in cropland on a decennial timescale [17-18].

Due to mineral 74 dissociation and decreased K balance in soil, Nepal is now experiencing a deficiency of K in 75 field crops [19-20]. Subsequently, the K consumption rate of Nepal in 2009 was 0.45 kg K2O 76 ha-1, which slightly increased in 2016 to 1.5 kg K2O ha-1; however, this is still far less than 4 the national recommended dose of 30 kg K2O ha-1 [21].

So, we considered 77 15 kg K2O ha-1 as 78 a basal recommended K dose (BRKD) in the current experiment [22]. 79 For instance, the yield of RWCS in recent decades is decreasing mainly due to a decline in 80 soil fertility [23-24]. Yields of RWCS are also declining due to the incidence of diseases such 81 as rusts, leaf blight, spot blotch [25],

and a change in temperatures in the region during the 82 grain filling period [26]. Many reports have indicated a decreasing trend in soil fertility, 83 including K concentration in soils of Nepal [19, 27].

A recent soil test report from soil testing 84 mobile van program in 9 (out of 75) districts in Nepal revealed that around 33% of total 85 sample tested (n = 1479) had soil K at 10-30 kg K2O ha-1 or even less [28]. Due to farmers' 86 use of farmyard manure (FYM), mineralized K from FYM is the primary K input in soil. The 87 current FYM application rate among Nepalese farmers is about 2.5-3 t ha-1 annually; 88 however, the inferior quality of manure (N – 0.5 to 0.8%, P – 0.2%, and K – 0.5%) [29] has 89 resulted in a very high FYM recommendation rate (20-30 t ha-1), which is far beyond farmers' 90 achievability [30-31].

Thus, mineral K sources are necessary to replenish the mined K from 91 cropping soils. 92 A proper nutrient management plan is necessary to sustain yield in the long run with a 93 revision of a possibly outdated K fertilizer rate. Improved productivity of RWCS in Nepal 94 with a revised rate of K fertilizer is also a major concern to keep up with population growth 95 in Nepal, which is predicted to be 36 million by mid-2050 [32-33]. Previous K 96 recommendations do not consider the environmental K response factor.

In this study, we 97 selected three agro-ecozones of Nepal to address this shortcoming. Because increasing K 98 levels in K deficient soils may increase crop productivity in RWCS, we aimed to study the 99 yield response and nutrient response of additional K fertilizer in rice and wheat in three 100 predominant RWCS agro-ecozones in Nepal: the inner-Terai, Terai, and high-Hills. 5 2 101 Materials and Methods 102 2.1

Study Area and Climate 103 Nepal is divided into five agro-ecozones (Terai, inner-Terai, mid-Hills, high-Hills, and high- 104 Himalayas). Elevation ranges from 80 meters above sea level (masl) in Terai to 8,848 masl 105 in the high-Himalayas. Cultivation is conducted up to the 4,800 masl high-Hills [34]. The 106 climate in all agro-ecozones is wet summer and dry winter subtropical.

Average annual 107 rainfall ranges from 1000-1800 mm and more than 80% of annual rainfall occurs during the 108 monsoon period, while the winter season is generally cold and humid with scarce rainfall 109 [35]. 110 This research was conducted in research stations at the Nepal Agricultural Research Council 111 (NARC), an apex government institution of agriculture research representing different agro112 ecozones of Nepal.

The research was the regular annual project of the Soil Science Division 113 (SSD), which was approved by the reviewing committee. All of the approved projects for 114

multi-location trial within NARC research stations were allowed to conduct field trials 115 collaborating with their respective research stations.

The first research site was established 116 in 2009-2010 at the National Maize Research Program, Rampur, Chitwan, which represents 117 the inner-Terai agro-ecozone situated at the coordinates 27°39' N, 84°20' E and an elevation 118 of 187 masl. The second research site was established in 2011-2012 at Hill Research Station, 119 Kabre, Dolakha, representing the high-Hills agro-ecozone with the coordinates 27°38' N, 120 86°9' E and an elevation of 1820 masl.

The third research site was established in 2012-2014 121 at the Regional Agriculture Research Station, Parwanipur, Bara, which represents the Terai 122 agro-ecozones with the coordinates 27°4' N, 84°55' E and an elevation of 96 masl. 123 Weather data was collected in the research station. Symon's rain gauze was used to 124 measure precipitation and a maximum-minimum thermometer was used to record the 6 temperature.

Daily weather parameters were recorded that 125 were then averaged over the 126 months and years. During the research period, the Kabre site received 2370 mm annual 127 precipitation with the maximum rainfall (790 mm) in August and the minimum (0 mm) 128 rainfall from December to February. The annual temperature ranged from 26? C (in May 129 and June) to 3? C (in January).

The average maximum and minimum temperatures were 22? 130 C and 12? C, respectively, for the region. Similarly, the Rampur site received 2290 mm 131 annual precipitation with the maximum rainfall (697 mm) in August and the minimum 132 rainfall (0 mm) in November and January. The annual temperature ranged from 37? C (in 133 April) to 10? C (in December and January).

The average maximum and minimum 134 temperatures were 31? C and 21? C, respectively. The Parwanipur site received 1423 mm 135 annual precipitation with the maximum rainfall (311 mm) in August and the minimum 136 rainfall (0 mm) from November to January and in April. The annual temperature ranged 137 from 37.5? C (in April) to 10? C (in January).

The average maximum and minimum 138 temperatures were 22? C and 12? C, respectively. 139 2.2 Soil 140 A baseline soil sampling from the top 20 cm depth was collected and analyzed for soil 141 texture, pH, organic matter (OM), total N, available P, and available K concentrations (Table 142 1).

Soil samples from the top 20 cm were again collected from experimental plots in 2012 143 (Kabre site), 2010 (Rampur site), and 2013 (Parwanipur site) to observe changes in pH, OM, 144 total N, available P, and available K concentrations before the planting of the main crop. 145 After the harvest of the crops, three soil samples from the top 20 cm of a plot were 146 composited by mixing and removing gravel, roots, undecomposed materials, and debris. 7 The soil was then air dried, ground to break up aggregates, and 147 run through a 2 mm sieve 148 before being stored and subjected to lab analysis.

149 Table 1: Initial Soil Parameter test results, safe nutrient range, and major K minerals of the research sites (safe 150 limit/ medium range values adopted from Soil Science Division, Khumaltar, NARC, Nepal). 151 Location Safe limit/ medium range Current result Major K minerals [13] High hills pH 6.50 – 7.50 6.20 Mica OM†, % 2.50 – 5.00 0.75 (1.30) Total N, % 0.10 – 0.20 0.07 Available P205 ‡, kg ha-1 13.00 – 25.00 47.52 (107.30) Available K_2O§, kg ha-1 49.00 – 125.00 100.83 (121.00) Soil texture Sandy loam Terai pH 6.50 – 7.50 5.8 Mica OM, % 2.50 – 50 1.27 (2.20) Total N, % 0.10 – 0.20 0.14 Available P205, kg ha-1 13.00 – 25.00 52.44 (118.00) Available K_2O, kg ha-1 49.00 – 125.00 50.83 (61.00) Soil texture Sandy loam Inner-Terai pH 6.5 – 7.5 6.20 Mica OM, % 1.50 – 3.00 0.63 (1.10) Total N, % 0.07 – 0.15 0.11 Available P205, kg ha-1 13.00 – 25.00 21.33 (48.00) Available K_2O, kg ha-1 49.00 – 125.00 137.50 (165.00) Soil texture Silty clay 152 †Conversion factor 1.72 was used to convert OC to OM (OM = 1.72 x OC); 153 ‡Conversion factor 2.25 was used to convert P to P_2O_5 (P_2O_5 = 2.25 x P); 154 §Conversion factor 1.20 was used to convert K to K_2O (K_2O = 1.20 x K).

155 Note: This conversion was conducted for rest of the soil parameters throughout the manuscript. 156 157 Soil chemical analysis followed a protocol developed by the SSD to analyze the soil. Soil pH 158 was determined in a 1:2 soil water ratio using electric pH electrodes.

Soil organic carbon 159 was determined by the wet digestion method as described by the Walkley-Black method. 160 The Kjeldahl method was used to determine total N in which soil is digested in concentrated 161 sulphuric acid, while available P was determined in a spectrophotometer as described by a 162 modified Olsen method. Available K from the soil was extracted in a 0.1 M ammonium 163 acetate solution and determined using a flame photometer.

8 2.3 164 Experimental Design and Treatment 165 At all three sites, the experimental design was laid out in a randomized complete block 166 design with four replications. Treatment included seven rates of K fertilizer as 0, 15, 30, 45, 167 60, 75 and 90 kg K_2O ha-1. We considered 15 kg K_2O ha-1 as basal recommended K dose 168 (BRKD). The N and P fertilizers were applied at a rate of 100 kg N ha-1 and 30 kg P_2O_5 ha-1 in 169

each plot.

Half rates of N, full rates of P, and full rates of K were applied as basal at the time 170 of transplanting and the remaining half rate of N was applied at the tillering stage. All of the 171 fertilizers were distributed and ploughed immediately during basal dose application in both 172 crops. 173 In this study, urea, triple superphosphate (TSP), and muriate of potash (MOP) were sources 174 of N, P, and K, containing 46% N, 46% P2O5, and 60% K2O, respectively.

No organic fertilizer 175 sources were used in the research plots. All of these fertilizers were sourced from Salt 176 Trading Corporation Limited, Kathmandu (coordinates - 27° 40' 39.77"N, 85° 21' 12.05" E; 177 fertilizer source origin – India; appearance of urea – white, crystals form, and solid state; 178 TSP – light brownish, granular form, and solid state; MOP – reddish, mixed crystal and 179 powder form, solid state).

Similarly, rice and wheat seeds were sourced from the Botany 180 Division of NARC and were the recommended varieties for the regions. The WK 1204 variety 181 of wheat and Ram Dhan variety of rice were selected for the high-Hill region and the Gautam 182 variety of wheat and Hardinath variety of rice were selected for the inner-Terai and Terai 183 regions.

Wheat seeds were directly sown in the field while 21 day-old rice seedlings were 184 transplanted in the puddled field in a row-to-row spacing of 20 cm and hill-to-hill spacing of 185 20 cm. 186 2.4 Statistical Analysis 9 Yield and yield parameters of rice and wheat were collected 187 during the growing seasons. 188 Length parameters (plant height and panicle length) were measured by using scale and 189 weight parameters (grain yield, straw yield, biomass, and thousand-grain weight) were 190 measured. Tiller number was recorded with manual counting.

Relative yield (RY) was 191 calculated by subtracting control yield from treatment yield (delta yield), divided by control 192 yield, and expressed as a percentage. ???? (%) = ?????????? ?????????? ?????????????? ?????????? 193 ?? 100 (Equation 1) 194 The maximum physical K rate was calculated by equating the first-order derivative of the 195 yield response function with zero and the economic optimum K rate was calculated by 196 equating the first-order derivative of the yield response function with the price ratio [36].

For maximum physical K rate, ???? ???? 197 = ?? (Equation 2) For economic optimum K rate, ???? ???? = ???? ???? 198 (Equation 3) 199 Where, ?y = yield response function, ?x = derivative for x, Py = unit price of fertilizer, 200 Px = unit price of grain yield. 201

Collected data were compiled and cleaned in MS Excel 2016. The assumption of analysis of 202 variance (ANOVA) was tested and means were separated at a 5% level of significance using 203 Tukey's test using R-studio (v. 1.3.1056).

Time (in years) and location were not combined to 204 analyze as fixed variables and were treated individually. A correlation test was carried out 205 between different parameters at a 95% level of significance. 206 3 Results 207 3.1 Soil chemical properties 208 In a year, changes in soil chemical properties due to the addition of different rates of K 209 fertilizer were measured after harvest of the first year's crop (in different years: Kabre in 10 2012, Rampur in 2010 and Parwanipur in 2013) at different 210 sites and their values are 211 presented in Supplemental Table S1. There were no significant changes in pH, OM, N, and 212 P (except at the Rampur site) in addition to K at different rates.

Soil K concentration 213 increased with addition of K rates at the Kabre site compared to the control. Only K rates at 214 60 kg K2O ha-1 or higher increased soil K concentration compared to the control at the 215 Rampur site (Supplemental Table S1). 216 3.2 Yield Response 217 3.2.1

Inner Terai (Rampur) 218 Thousand-grain weight, grain yield, and straw yield of rice and wheat increased with the 219 addition of K fertilizer in the inner-Terai agro-ecozone (Table 2). Results showed an 220 increased demand for K fertilizer to obtain a significantly higher yield of rice than the BRKD 221 (15 kg K2O ha-1). A potassium fertilizer rate of at least 45 kg K2O ha-1 or higher produced 222 significantly greater yields, with a trend of r2 = 0.69, P = 0.122 in 2009, r2 = 0.55, P = 0.26 in 223 2010 and r2 = 0.94, P = 0.006 in 2011.

Similarly, a significantly higher rice straw yield was 224 obtained from 75 kg K2O ha-1 than the BRKD in the first and third years. However, rice yield 225 declined up to 30-33% in the second year (Table 2). Table 2. Analysis of variance (ANOVA) results with means for thousand-grain weight, grain yield, and straw yield of rice as affected by different 226 rates of potassium 227 fertilizer in a rice-wheat cropping system at NMRP, Rampur, Chitwan, Nepal over three consecutive years.

Treatment† Thousand-grain weight, g Grain yield, kg ha-1 Straw yield, kg ha-1 2009 2010 2011 2009 2010 2011 2009 2010 2011 0 19.0c‡ 21.9 19.2b 2210.0c 1978.0c 2102.0c 2762.0c 2419.0b 2392.0c 15 19.5b 21.7 19.6ab 4439.0b 3259.0b 3010.0b 5256.0b 4056.0a 4032.0b 30 20.0ab 20.1 19.9ab 4818.0ab 3609.0ab 3219.0ab 6250.0ab 3903.0a 4541.0ab 45 19.8b 20.6 19.8ab 4960.0a 3502.0ab 3129.0ab 6037.0ab 3944.0a 4538.0ab 60 20.0ab 22.1 20.3a 5161.0a 3875.0a 3289.0ab 6002.0b 4376.0a 4335.0ab 75 20.5a 21.9 20.3a

5241.0a 3641.0ab 3555.0a 7067.0a 4125.0a 4862.0a 90 19.6b 22.2 20.0a 4890.0a 3576.0ab 3569.0a 6188.0ab 3861.0a 4496.0ab Significance *** NS * *** *** *** *** ** *** 228 †Treatments included 0 (or control), 15, 30, 45, 60, 75, and 90 kg K2O ha-1.

229 ‡Means in a column followed by the same lowercase letter are not significantly different. 230 *P < 0.05,* *P < 0.01, ***P < 0.001, and NS = not significant. Thousand-grain weight (TGW) of rice was significantly increased compared 231 to the BRKD in 232 the first and third years from 75 and 60 kg K2O ha-1, but no significant increment was 233 observed in the second year (Table 2).

Plant height was affected by K rate 75 kg K2O ha-1 234 and significantly higher than the BRKD, but panicle length and tiller number did not differ 235 significantly with the BRKD (Supplemental Table 2). All of these yield-attributing traits were 236 positively correlated with yield, but a strong positive correlation (r2 = 0.87, p = 0.000) was 237 reported with plant height (Supplemental Table 5).

238 Wheat grain and straw yield significantly increased compared to the BRKD with an 239 additional level of K (Table 3). The significant yield of wheat increased from 30 to 75 kg K2O 240 ha-1 in the first and second year, respectively A subsequent decline of 38% in grain yield and 241 20% in straw yield was observed over the three years (Table 3). Table 3.

ANOVA results with means for thousand-grain weight, grain yield, and straw yield of wheat as affected by different rates of potassium 242 fertilizer in a rice-wheat 243 cropping system at NMRP, Rampur, Chitwan, Nepal over three consecutive years. Treatment† Thousand-grain weight, g Grain yield, kg ha-1 Straw yield, kg ha-1 2009 2010 2011 2009 2010 2011 2009 2010 2011 0 42.5cd‡ 25.6f 26.4d 961.0d 352.0d 533.0d 2663.0c 1005.0d 1674.0c 15 40.7d 31.2e 32.1c 1930.0c 895.0c 1844.0c 6001.0b 1705.0d 4380.0b 30 44.5bcd 39.8b 35.8b 3504.0b 1843.0b 2289.0b 6924.0ab 3054.0bc 4843.0ab 45 45.5bc 38.1c 37.7ab 4025.0ab 1633.0b 2882.0a 7066.0ab 2734.0c 5093.0ab 60 51.2a 34.4d 39.3a 4190.0ab 2442.0a 2761.0a 7102.0ab 3796.0ab 5840.0a 75 48.5ab 43.5a 35.7b 4529.0a 2562.0a 2628.0ab 7137.0ab 3888.0a 5591.0a 90 53.0a 37.5c 37.1ab 4374.0a 2288.0a 2853.0a 7244.0a 3579.0ab 5520.0a Significance *** *** *** *** *** *** *** *** *** 244 †Treatments included 0 (or control), 15, 30, 45, 60, 75, and 90 kg K2O ha-1.

245 ‡Means in a column followed by the same lowercase letter are not significantly different. 246 ***P < 0.001 and NS = not significant. 247 248 249 Thousand-grain yield was significantly increased at 60 kg K2O ha-250 1 compared to the BRKD. 251 Plant height was significantly higher than the BRKD, with the highest plant height reported 252 from the 90 kg K2O ha-1 dose in the first year and the 75 kg K2O ha-1 dose in the

second year 253 (Table 3).

There was no significant increment in panicle length and tiller number with the 254 BRKD and no significant increment in tiller number with the control in the second and third 255 years (Supplemental Table S4). The highest positive correlation was observed between 256 wheat grain yield and plant height ($r2 = 0.82$, $p = 0.000$) and panicle length ($r2 = 0.74$, $p =$ 257 0.000) (Supplementary Table S5). 258 3.2.2

High Hills (Kabre) 259 The additional K rate had no significant response over the BRKD in straw yield and grain 260 yield of wheat in both years and of rice in the first year (Table 4). Rice grain and straw yield 261 were significantly increased in the second year of potassium application with 30 kg $K2O$ ha- 262 1. Over the two years, grain yield of rice increased by 10%, but straw yield declined by 22%. Table 4.

ANOVA results with means for thousand-grain weight, grain yield, and straw yield of rice and wheat as affected by different rates 263 of potassium fertilizer in a 264 rice-wheat cropping system at HRS, Kabre, Dolakha, Nepal over two consecutive years. Rice Wheat (2011) Treatment† Thousand-grain weight, g Grain yield, kg ha-1 Straw yield, kg ha-1 Thousand-grain weight Grain yield, Straw yield, 2010 2011 2010 2011 2010 2011 (g) (kg ha-1) (kg ha-1) 0 19.12 20.1 1783.0b 1941.0c 3870.0b 2815.0c 51.9 890.0b 3016.0b 15 17.93 18.6 2937.0a 2958.0b 8508.0a 5181.0b 54.6 2416.0a 5831.0a 30 18.22 19.1 3408.0a 3254.0a 9266.0a 6154.0ab 55.4 2422.0a 6988.0a 45 18.53 19.2 3272.0a 3187.0ab 8846.0a 6129.0ab 54.1 2461.0a 5994.0a 60 18.05 18.7 3017.0a 3198.0ab 9043.0a 6092.0ab 51.6 2634.0a 6923.0a 75 18.03 18.7 3325.0a 3297.0a 9053.0a 6624.0a 53.6 2215.0a 6236.0a 90 18.4 19.4 3459.0a 3256.0a 8367.0a 6019.0ab 53.3 2607.0a 6111.0a Significance NS NS *** *** *** *** NS *** ** 265 †Treatments included 0 (or control), 15, 30, 45, 60, 75, and 90 kg $K2O$ ha-1.

266 ‡Means in a column followed by the same lowercase letter are not significantly different. 267 ** $P < 0.01$, ***$P < 0.001$, and NS = not significant Thousand-grain weight of rice did not significantly increase in either 268 the first or second year 269 (Table 4). Plant height of rice significantly increased at the 45 kg $K2O$ ha-1 application in the 270 second year, but tiller number and panicle length in rice, and plant height and tiller number 271 in wheat were not significantly different compared with the BRKD (Supplemental Table S6).

272 Panicle length of wheat was significantly increased at 60 kg $K2O$ ha-1 compared to the BRKD. 273 A significant correlation of grain yield was observed with plant height of rice ($r2 = 0.81$, $p =$ 274 0.000), plant height of wheat ($r2 = 0.68$, $p = 0.000$), and tiller number ($r2 = 0.75$, $p = 0.000$) 275 (Supplemental Tables S7 and S8). 276 3.2.3

Terai (Parwanipur) 277 Grain yield and straw yield significantly increased compared to the control but not with the 278 BRKD in rice (Table 5). However, a significant response of additional K fertilizer was reported 279 from 60 Kg K2O ha-1 in wheat. An incremental yield of rice was achieved up to 60 kg K2O ha- 280 1 but declined afterward.

Over the two years, yield increased up to 30% and incremental 281 highest yield over control treatment was reported up to 80%. The addition of K did not 282 increase the thousand-grain weight of rice in either year. A 33% increase in wheat grain 283 yield was reported from 60 kg K2O ha-1 compared to the BRKD. Wheat straw yield was 284 similar (3.7

t ha-1) at 60 kg K2O ha-1 in both years. Table 5. ANOVA results with means for thousand-grain weight, grain yield, and straw yield of rice and wheat as affected by different rates 285 of potassium fertilizer in a 286 rice-wheat cropping system at RARS, Parwanipur, Bara, Nepal.

Rice Wheat Treatment† Thousand-grain weight, g Grain yield, kg ha-1 Straw yield, kg ha-1 Thousand-grain weight, g Grain yield, kg ha-1 Straw yield, kg ha-1 2013 2014 2013 2014 2012 2014 2013 2014 2013 2014 2013 2014 0 19.9 22.6 1507.0b‡ 1551.0c 401.0b 3953.0b 41.7b 45.5b 761.0b 1029.0d 1196.0d 1282.0d 15 22.1 22.1 2287.0a 2993.0ab 6823.0a 6691.0a 43.3b 46.3b 2474.0a 2452.0c 3241.0c 2746.0c 30 21.1 21.6 2423.0a 2997.0ab 6351.0a 7492.0a 45.9a 47.2ab 2614.0a 2777.0bc 3472.0bc 3170.0b 45 21.5 23.2 2095.0a 2344.0b 6702.0a 6018.0a 46.3a 44.8b 2475.0a 2829.0abc 3412.0bc 3211.0b 60 22.5 23.4 2623.0a 3333.0a 6707.0a 7104.0a 47.7a 46.9b 2767.0a 3310.0a 3770.0a 3776.0a 75 22.9 23.1 2092.0a 2772.0ab 6827.0a 6778.0a 47.4a 47.3ab 2633.0a 3055.0ab 3655.0ab 3627.0a 90 23.3 22.9 2159.0a 3244.0a 6917.0a 7160.0a 47.3a 50.3a 2556.0a 3038.0ab 3865.0a 3431.0ab Significance NS NS ** *** *** *** *** * *** *** *** *** 287 †Treatments included 0 (or control), 15, 30, 45, 60, 75, and 90 kg K2O ha-1.

288 ‡Means in a column followed by the same lowercase letter are not significantly different. 289 *P < 0.05,* *P < 0.01, ***P < 0.001, and NS = not significant. The thousand-grain yield of wheat was significantly higher from 30 kg 290 K2O ha-1 in the first 291 year and 90 Kg K2O ha-1 in the second year compared to the control (Table 5). In both crops 292 yield attribute traits other than tiller number (i.e.,

plant height and panicle length) 293 significantly increased compared to the control but were similar to the BRKD. Significantly, 294 the highest tiller number of both rice (260 m-2) and wheat (312 m-2) was obtained from 90 295 kg K2O ha-1 (Supplemental Table

S9). Strong positive significant correlation exists between 296 yield and yield-attributing traits (plant height and panicle length) of rice (r2 = 0.82, p = 0.000) 297 and wheat (r2 = 0.76, p = 0.000), though a weak non-significant correlation (r2 = 0.32, p = 298 0.87) exists with thousand-grain weight (Supplemental Tables S10 and S11). 299 3.3 Nutrient Response 300 3.3.1

Physical Maximum and Economic Optimum Rates 301 Physical maximum and economic optimum rates of K for rice and wheat at all agro ecozones 302 were near values (Table 6). The rate of K for physical maximum production was highest for 303 wheat in inner-Terai at 4.8 times the recommended rate, and was the least for rice in Terai 304 at 3.4 times the recommended rate.

Similarly, the K requirement for wheat should be 305 increased by 4.7 times in inner-Terai and 3.9 times in the high-Hills and Terai. 306 2 Table 6. Physical maximum rate and economic optimum rate of potassium 307 fertilizer recommended 308 for the rice-wheat cropping system in different agro-ecozones of Nepal. Agro-ecozone Crop Nutrient response curve function Physical maximum rate Economic optimum rate kg ha-1 High-Hills (Dolakha) Rice y = -0.4627x2 + 58.401x + 2397.1

68.0 67.0 Wheat y = -0.5017x2 + 72.826x + 634.0 60.0 58.0 Inner-Terai (Chitwan) Rice y = -0.2535x2 + 30.839x + 1812.2 63.0 57.0 Wheat y = -0.4946x2 + 60.738x + 1197.4 73.0 71.0 Terai (Bara) Rice y = -0.3245x2 + 41.085x + 2121.3 51.0 50.0 Wheat y = -0.3983x2 + 47.665x + 1255.3 61.0 59.0 309 y = yield obtained (dependent variable) and x = fertilizer dose (independent variable).

The nutrient response 310 curve function was derived based on the averaged data over the years of the respective locations. 311 3.3.2 Expected Yield and Relative Yield over Residual Fertility Yield 312 Additional K rates increased the relative yield of rice and wheat over soil inherent/residual 313 fertility (yield obtained from the control plot) with incremental expected yield (Figure 1).

314 The residual fertility rice yield is around 2 t ha-1 in all agro-ecozones. The increased K rate 315 was expected to increase the yield of rice maximally by 1.8, 0.9, and 1.3 t ha-1 in inner-Terai, 316 Terai, and high-Hills, respectively. Similarly, the residual fertility yield of wheat was 0.6 t ha- 317 1 in inner-Terai and around 1 t ha-1 in Terai and high-Hills. The increased K rate was expected 318 to increase the yield of wheat maximally by 2.6, 1.8, and 1.4 t ha-1 in inner-Terai, Terai, and 319 high-Hills, respectively. The response of K was high in inner-Terai due to the low residual 320 potassium level.

321 322 323 [Figure 1] 324 Figure 1. Aggregated data analysis to compare the different

levels of potassium application on expected yield 325 (represented by the bar graph) and relative yield (represented by the green line graph) over residual fertility 326 yield (represented by the straight blue line) in rice (Figure 1A, 1C, 1E) and wheat (Figure 1B, 1D, 1F) in inner- 327 Terai (1A, 1B), Terai (1C, 1D), and high-Hills (1E, 1F).

3 328 4 Discussion 329 Increased yields of rice and wheat in the inner Terai and Terai regions of Nepal with K 330 fertilizer application rates of 50 to 60 kg K2O ha-1 make it evident that these regions have 331 low availability of nutrients due to sandy soil, high rainfall intensity, and frequent leaching 332 loss of K [20]. Other findings also reported low availability of K in these regions [5, 37-38].

333 The soils of these regions are generally characterized as fertile soil, which is made up of 334 recent alluvial deposits, mostly fine sand and silt with light to medium texture, with the 335 Terai region of Nepal considered the basket of grain [39-40]. Crop cultivation in the Terai 336 region began after the clearing the forest in 1927 [41], and robust agricultural production 337 was observed with the alluvial deposit, medium-textured, forest soils.

In later decades, 338 however, low organic input, nutrient removal due to crop harvesting, heavy tillage work, 339 continuous erosion, and poor crop and land management resulted in a low nutrient reserve 340 [42-43]. Hence, K, an active component of the nutrient cycle, is one of those nutrients 341 impacted by low nutrient reserve. 342 In high-Hills, increased crop yields were recorded with additional K rates in the second year 343 starting at 30 kg K2O ha-1 up to 70 kg K2O ha-1.

Soil in the region is low in OM content, with 344 a high erosion rate and sandy loam texture with acidic soil pH [44-45]. Additionally, the mid345 and-high-Hills are characterized by an erosion rate of 32-38 t ha-1 yr-1 [46]. Carson [47] 346 reported that a 1 mm loss of topsoil was estimated to cause a loss of 10 kg N ha-1, 15.7 kg 347 P2O5 ha-1, and 22.5 kg K2O ha-1.

Bijay-Singh and Vashishta [48] reported that for the 348 production of rice and wheat at 8 to 10 t ha-1, it can remove around 250 kg K2O ha-1 from 349 the soil. In contrast, rice-growing farmers in the mid-Hill regions have benefited from the 350 accumulation of eroded sediments (for example, mica), a source of exchangeable K [49-51]. 351 Additionally, soil pH is a limiting factor for K availability in the high-Hills (Supplemental Table 4 1).

The response of additional K fertilizer compared to the 352 BRKD did not significantly 353 increase in wheat and rice yields (Table 4) because the acidic pH (4.4) might restrict

the 354 availability of K, as microbial nutrient transformation is restricted below a soil pH of 5.5 [52]. 355 Comparing the K fertilizer consumption under RWCS in the IGP region, Nepal uses less K 356 fertilizer in rice (0.9 kg K2O ha-1) and wheat (2.0

kg K2O ha-1) than its neighbors. India uses 357 9.4 kg K2O ha-1 in rice and 4.6 kg K2O ha-1 in wheat, whereas Bangladesh uses 9.9 kg K2O ha- 358 1 in rice and 7.9 kg K2O ha-1 in wheat [2]. In Asia, China has the highest K use rate in both 359 rice (33.2 kg K2O ha-1) and wheat (26.6 kg K2O ha-1). A long-term rice-wheat fertility 360 experiment conducted in Bhairahawa, Nepal showed that annual K balance was negative 361 and that K exhibited a significant response in rice [5]. The increase in yield of both rice and 362 wheat was evident in all three agro-eco zones of Nepal.

363 Nutrient response functions showed that the physical and economic limits of K rate were 364 more or less equal at all locations. The K requirement of rice should be increased by 4.4, 365 3.8, and 3.3 times BRKD in high-Hills, inner-Terai, and Terai, respectively. In the current 366 study, the economic optima of K ranged from 50 to 67 kg K2O ha-1 in rice and 58 to 71 kg 367 K2O ha-1 in wheat in different locations.

The availability of K in soil is proportional to the 368 added K fertilizer [53], but a higher rate of K addition results in fixation rather than 369 availability [50]. As potassium is a so-called luxury consumption nutrient, additional K rates 370 can sometimes result in loss [54]. In addition, K is a mobile element and additional K results 371 in leaching under flooded conditions in the case of rice cultivation [52].

Thus, the current 372 study was unable to attain a more economic yield with 90 kg K2O ha-1, meaning that it is not 373 necessary to apply more than 70 kg K2O ha-1, starting from 50 kg K2O ha-1 depending on the 374 crop type and agro-eco zone. 5 At all locations, additional K rates increased relative yield between 40 375 to 50 and 60 to 80% 376 in rice and wheat.

Rice yield was estimated to increase up to 3 to 4.5 t ha-1 and wheat yield 377 was estimated to increase 2.5 to 3 t ha-1 from 60 kg K2O ha-1. This different K response in 378 rice and wheat is observed due to the K limitation in the soil and the availability of K after K 379 fertilizer application. The soils of the studied region were high in mica content, and in soils 380 containing high mica, even 1 to 2% of total K is enough.

However, continual crop removal 381 of K and restricted K application in soil for long periods resulted in the weathering of mica 382 into biotite or vermiculite, an avenue for K limitation [55]. Similarly, the exchange of K is 383 possible between available and fixed pools. More than 50% of total K availability in RWCS is 384 obtained from fixed K pools

that may further deplete the K reserve from the soil [38].

Thus, 385 it is imperative to apply K fertilizer to maintain K levels and soil fertility in the cropland. 386 Soil N and P are also known to affect K availability and uptake by plants. Application of 387 nitrogen and phosphorus are reported to have resulted in a 145% increase in K uptake 388 compared to a control [56] and Tiwari et al.

[57] reported that response to K application in 389 rice increased with increasing rates of N application. Under nitrogenous fertilization, interlayer K can also be replaced by NH4 390 +, as both have a nearly similar ionic size, influencing 391 availability. The current study reported that there were no differences in soil total N after a 392 year’s addition of K fertilizer, suggesting no adverse effects on total N due to treatment 393 application.

These days, Nepalese farmers are aware of the importance of chemical 394 fertilizers in crop production and the application of chemical fertilizers is gradually 395 increasing. Most farmers use only N-related fertilizers, which may allow them to reach 396 potential yields. However, this can increase the cost of cultivation in the short term and 397 cause reduction in soil quality and productivity in the long term.

Because a continuous and 398 increased application of N fertilizer is not enough to replenish lost plant nutrients and 6 maintain soil productivity [27, 58], a balanced fertilization 399 is necessary to increase the 400 productivity of RCWS. 401 To summarize, the current study recommends revising the current recommended dose (30 402 K2O ha-1) to 60 to 70 kg K2O ha-1 in different agro-eco zones.

The use of a single variety of 403 rice and wheat, which was suitable to the soils of research sites, could be a potential pitfall 404 of this study, while the K rate might differ for hybrid cultivars of rice and wheat and different 405 soil types in different agro-ecozones. So, a series of experiments in other agro-ecozones are 406 suggested using different varieties of crops in different soil types and rotations.

407 5 Conclusion 408 Improvements in crop yield and soil nutrient response with the addition of K fertilizer in 409 RWCS at three agro-ecozones of Nepal suggests that the current recommended rate of K 410 fertilizer (30 kg K2O ha-1) should be increased about 1.5 to 2 times for rice and 2 to 2.5times 411 for wheat to achieve optimum economic production.

These recommendations are made for 412 the improved crop varieties with a yield potential of 2 to 4 t ha-1 of rice and wheat, which 413 may vary for other high-yielding

varieties. Fertilizer recommendation is a dynamic and 414 continuous process that largely depends on soil type, crop response, inherent fertility, grain 415 to fertilizer price ratio, and environmental factors, and recommendations should be revised 416 regularly over time while considering these factors.

K, an integral crop nutrient component 417 that contributes to soil fertility and optimum crop production, should be applied at the 418 recommended rate in a balanced way by appraising available K sources.

INTERNET SOURCES:

<1% - https://mafiadoc.com/soil-fertility-and-plant-nutrient-management_5c1ef5ad097c4772298b45b1.html
<1% - https://mafiadoc.com/session-11-soil-and-residue-management_5a4f96ed1723dd0243970a40.html
<1% - https://www.researchgate.net/profile/Yashbir_Shivay2
<1% - https://www.ncbi.nlm.nih.gov/pmc/articles/PMC6183874/
<1% - https://onlinelibrary.wiley.com/doi/full/10.1002/jpln.202000020
<1% - https://www.slideshare.net/AndrewGates/dynamics-of-k-in-soils-and-their-role-in-management-of-k-nutrition
<1% - https://www.researchgate.net/publication/325488444_Phosphorus_Nutrition_Plant_Growth_in_Response_to_Deficiency_and_Excess
<1% - https://www.researchgate.net/publication/283338092_Grain_yield_water_productivity_and_CH4_emission_of_irrigated_rice_in_response_to_water_management_in_south_China
<1% - https://www.researchgate.net/publication/26542000_Effect_of_different_sources_and_rates_of_nitrogen_and_supra_optimal_level_of_potassium_fertilization_on_growth_yield_and_nutrient_uptake_by_sugarcane_grown_under_saline_conditions
<1% - https://www.researchgate.net/publication/224902400_Soil_Organic_carbon_fractions_after_16-years_of_applications_of_fertilizers_and_soil_organic_manure_in_a_Typic_Rhodalfs_in_semi_arid_tropics
<1% - https://www.researchgate.net/publication/222193652_Trends_in_maize_rice_and_wheat_yields_for_188_nations_over_the_past_40_years_A_prevalence_of_linear_growth

<1% -
https://www.researchgate.net/publication/301284566_Sustainability_issues_on_rice-whe
at_cropping_system
<1% -
https://www.cambridge.org/core/journals/plant-genetic-resources/article/longitudinal-a
nalysis-of-maize-diversity-in-yucatan-mexico-influence-of-agroecological-factors-on-la
ndraces-conservation-and-modern-variety-introduction/CAB4EA86B65C7615436B4656F
09E65BB
<1% -
https://mafiadoc.com/cold-water-fisheries-in-the-trans-himalayan-countries-ftp-directo
ry-_5a3561cf1723ddfd577f92ba.html
<1% -
https://newlifeinburiedbones.wordpress.com/2015/05/04/physiological-and-evolutionar
y-mechanisms-of-fertility-at-high-altitude-part-1/
<1% - https://agupubs.onlinelibrary.wiley.com/doi/full/10.1029/2009JF001426
<1% - https://csisa.org/category/csisa-success-story/nepal/page/2/
<1% -
https://www.researchgate.net/publication/330405150_Genetic_Variability_and_Correlatio
n_Coefficients_of_Major_Traits_in_Early_Maturing_Rice_under_Rainfed_Lowland_Environ
ments_of_Nepal
<1% - https://www.aplustopper.com/climate-india-icse-solutions-class-10-geography/
<1% - https://en.wikipedia.org/wiki/Temperature_in_Canada
<1% - https://www.ncdc.noaa.gov/sotc/global/201003
<1% - https://www.sciencedirect.com/science/article/pii/S1161030116301551
<1% - https://nepis.epa.gov/Exe/ZyPURL.cgi?Dockey=2000OL61.TXT
<1% -
https://www.scribd.com/document/369592107/Journal-of-Maize-Research-and-Develop
ment-Vol-2-No-1-2016
<1% -
https://www.slideshare.net/Guru6005/technologies-for-improving-grain-quality-of-cere
als
<1% -
https://www.researchgate.net/publication/228846969_Quantification_of_Organic_Carbon
_in_Soils_A_Comparison_of_Methodologies_and_Assessment_of_the_Carbon_Content_of
_Organic_Matter
<1% - https://www.rollitup.org/t/understanding-and-calculating-nutrient-ppm.995409/
<1% -
https://journals.lww.com/soilsci/Fulltext/2001/01000/OXIDIZIBLE_ORGANIC_CARBON_FR
ACTIONS_AND_SOIL.9.aspx
<1% - https://nepis.epa.gov/Exe/ZyPURL.cgi?Dockey=300009QJ.txt

<1% - https://www.sciencedirect.com/science/article/pii/S0048969720357181
<1% - https://www.researchgate.net/publication/240547096_Nutrient_Uptake_and_Apparent_Balances_for_Rice-Wheat_Sequences_II_Phosphorus
<1% - http://ijagbio.com/pdf-files/volume-6-no-3-2017/153-162.pdf
<1% - https://www.sciencedirect.com/science/article/pii/S0378429019300073
<1% - https://www.researchgate.net/publication/289918995_Integrated_nutrient_management_for_sugarcane_Saccharum_spp_hybrid_complex_plant-ratoon_system
<1% - https://www.sciencedirect.com/science/article/pii/S0016706118309212
<1% - https://www.sciencedirect.com/science/article/pii/S0304423818301961
<1% - https://www.investopedia.com/terms/d/dividendyield.asp
<1% - https://www.sciencedirect.com/science/article/pii/S0168169914001227
<1% - https://www.hindawi.com/journals/bmri/2014/841892/
<1% - https://paperity.org/p/47312777/using-chromosome-introgression-lines-to-map-quantitative-trait-loci-for-photosynthesis
<1% - https://journals.plos.org/plosone/article?id=10.1371/journal.pone.0127345
<1% - https://www.researchgate.net/publication/315746813_Continuous_Variables_Analysis_of_Variance
<1% - http://pubs.sciepub.com/wjar/2/4/6/
<1% - https://www.sciencedirect.com/science/article/pii/S026121941930287X
<1% - http://www.scdcpndabuleni.ro/wp-content/uploads/Lucrari-?tiin?ifice-CCDCPN-Dabuleni-Vol-XIX.pdf
<1% - https://www.mdpi.com/1660-4601/17/20/7459/htm
<1% - https://onlinelibrary.wiley.com/doi/full/10.1111/mcn.12794
<1% - http://cropresearch.org/volume-12-number-2-august-2011/
<1% - https://www.sciencedirect.com/science/article/pii/S0167198720305547
<1% - https://www.sciencedirect.com/science/article/pii/S1672630817300525
<1% - https://www.scielo.br/scielo.php?script=sci_arttext&pid=S0100-83582015000100023
<1% - https://academic.oup.com/jee/article/113/4/1741/5826482
<1% - https://www.thefreelibrary.com/EFFECT+OF+LONG+TERM+FERTILIZATION+ON+GRAIN+YIELD+AND+YIELD+COMPONENTS...-a0546690870
<1% - https://cropresearch.org/volume-18-no-2-june-2017/
<1% - http://aos.ro/wp-content/anale/AVol1Nr1Art.6.pdf
<1% - https://jitc.bmj.com/content/8/2/e000473

<1% -
https://www.slideshare.net/ijtra/biochemical-changes-of-nutrients-in-rice-plant-under-s
odic-soils
<1% -
https://www.researchgate.net/publication/331562864_Growth_yield_and_nitrogen_uptak
e_in_rice_crop_grown_under_elevated_carbon_dioxide_and_different_doses_of_nitrogen_
fertilizer
<1% - http://article.sapub.org/10.5923.j.ijaf.20201002.02.html
<1% -
https://www.researchgate.net/publication/229344281_A_review_of_resource_conserving_
technologies_for_sustainable_management_of_the_rice-wheat_cropping_systems_of_the_
Indo-Gangetic_plains_IGP
<1% -
https://www.researchgate.net/publication/323551916_EFFECTS_OF_ZINC_BORON_AND_
SULPHUR_ON_THE_YIELD_AND_NUTRIENTS_UPTAKE_OF_WHEAT_CROP
<1% - https://mafiadoc.com/progressive-horticulture_5c1f0b8f097c47641c8b4578.html
<1% -
https://www.lsuagcenter.com/~/media/system/1/2/4/b/124b05f050bcff362b8de6a201f9
5bd3/6%20weed%20control%20section%202015pdf.pdf
<1% - https://www.sciencedirect.com/science/article/pii/S0038071710004529
<1% - http://krishikosh.egranth.ac.in/handle/1/5810049752
<1% - https://www.frontiersin.org/articles/10.3389/fpls.2016.00643/full
<1% -
https://www.researchgate.net/publication/236594021_Effect_of_Integrated_Nutrient_Ma
nagement_on_Yield_Microbial_Population_and_Changes_in_Soil_Properties_under_Rice-_
Wheat_Cropping_System_in_Sodic_Soil
<1% - https://www.scirp.org/journal/PaperInformation.aspx?PaperID=75558
<1% -
https://www.researchgate.net/publication/306379153_Yield_response_of_wheat_to_nitro
gen_and_potassium_fertilization
<1% - https://www.frontiersin.org/articles/10.3389/fpls.2020.01307/full
<1% -
https://www.researchgate.net/publication/281645339_Long-term_soil_fertility_trial_in_ric
e-wheat_system_in_regional_agricultural_research_station_Khajura_Banke_results_of_soil_
analysis_data_from_1998_to_2006_and_2014_AD
<1% - https://researcherslinks.com/search/journals/%20Growth
<1% - https://www.sciencedirect.com/science/article/pii/S088557651630145X
<1% - https://www.sciencedirect.com/science/article/pii/S0168945218314286
<1% - https://onlinelibrary.wiley.com/doi/full/10.1002/pon.4878
<1% - https://pubs.acs.org/doi/10.1021/acs.est.9b07019

<1% -
https://www.researchgate.net/publication/317804535_Available_soil_macro_and_micro-nutrients_under_ricewheat_cropping_system_in_district_Tarn_Taran_of_Punjab_India
<1% - https://www.sciencedirect.com/science/article/pii/S037837741530086X
<1% -
https://www.entrepreneurindia.co/book-details/175/handbook-on-rice-cultivation-and-processing
<1% - https://issuu.com/adbevaluation/docs/ies-nep-final
<1% -
https://www.researchgate.net/publication/295252604_Impact_of_tillage_erosion_on_water_erosion_in_a_hilly_landscape
<1% - http://doczz.net/doc/1967062/proceedings-of-the-18th-nitrogen-workshop
<1% - http://doczz.net/doc/1161952/proceedings---18th-ciec-symposium
<1% -
https://www.researchgate.net/publication/315368642_Provincial_potassium_balance_of_farmland_in_China_between_1980_and_2010
<1% - https://mafiadoc.com/nepal_5c5a0ced097c4750088b459c.html
<1% -
https://www.researchgate.net/publication/285742474_Biophysical_and_economic_limits_to_negative_CO2_emissions
<1% -
https://www.researchgate.net/publication/287684372_Flaxseed_response_to_N_P_and_K_fertilization_in_South_Central_Chile
<1% -
https://www.researchgate.net/publication/269097984_Effect_of_potassium_and_zinc_fertilizer_on_crop_yield_nutrient_uptake_and_distribution_of_potassium_and_zinc_fractions_in_Typic_Ustipsamment_GAJANAND_JAT1_S_P_MAJUMDAR2_N_K_JAT3_and_SONALI_P_MAZUMDAR4
<1% - https://nepis.epa.gov/Exe/ZyPURL.cgi?Dockey=60000CWA.TXT
<1% -
https://www.researchgate.net/publication/312597590_A_practical_soil_management_to_improve_soil_quality_by_applying_mineral_organic_fertilizer
<1% - https://www.sciencedirect.com/science/article/pii/S0016706120313215
<1% - https://www.hindawi.com/journals/aess/2019/8764162/
<1% - https://pubs.acs.org/doi/10.1021/acsomega.9b04155
<1% -
https://www.sciencedirect.com/topics/earth-and-planetary-sciences/high-yielding-variety
<1% -
http://www.scielo.br/scielo.php?script=sci_arttext&pid=S1517-83822011000200018
