# Supplementary material for: Potassium nutrient response in the rice-wheat cropping system in different agro-ecozones of Nepal
Source: PLoS One. 2021 Mar 18;16(3):e0248837. doi: 10.1371/journal.pone.0248837 (PMC7971854; doi:10.1371/journal.pone.0248837)
Supplement: Attachment — Submitted filename: Review Notes_Nepal.docx [file pone.0248837.s002.docx]

Rice-Wheat cropping system is a wide spread cultivation system and often considered as one of the oldest known to mankind and we appreciate the author’s effort to execute a revised recommendation of Potassium for Rice-Wheat cropping system under a depleting soil nutrient scenario of different agro ecosystems of Nepal. As this manuscript contains a range of information of different aspects, we feel some re-arrangements and revisions may be beneficial for better comprehension of the content. These may be as follows-

A. In **abstract** of the manuscript there are mentions of “**1.** 45 to 75 kg K_2_O ha^-1^ in inner-Terai and Terai and 45 to 60 kg ha^-1^ in high-Hills conditions produced significantly higher grain yields compared to the RDKF.

**2.** A maximum rate of K fertilizer should not exceed 68 kg K_2_O ha^-1^ for rice in all agro-ecozones, 73 kg K_2_O ha^-1^ for wheat in inner-Terai and 60 kg K_2_O ha^-1^ for wheat in high-Hills and Terai as an **economically optimum** rate of K fertilizer for crop production.”

Here these two sentences seems to contradict each other in terms of amount of doses in different areas as the first sentence describes K doses for a generalized term “grain yields”, without any specific mention of rice or wheat. Whereas, highest limit mentions of crop specific K doses in the second sentence doesn’t match with the first one.

We will be grateful to receive a mention of Benefit: Cost ratio (BCR) (alongside values for physical and economic optimality) in the **Result** and **Discussion** that can potentially explain the *economical optimality* of the said doses in the **Abstract**, along with necessary corrections and/or explanations. Please also mention notations for ‘y’ and ‘x’ in the equations of **Table 5.**

B. In first part of **Introduction** Winter season of Wheat cultivation has been mentioned as “cold” and “***humid***”, whereas in **Materials and Methods**, Winter season has been described as “…generally ***dry*** *with* ***scanty rainfall***.” This also seems to be contradictory and needs necessary corrections.
C. We will be grateful to have K% in FYM used by farmers in Nepal and bibliographic reference of *occurrence of K deficiency in field crops in Nepal*; as mentioned in the **Introduction**.
D. We would like to know if there is any application of organic matter in this trial (as application of OM increases nonexchangeable K availability in soil (Pannu *et. al.*, 2003) and would appreciate if Organic Matter (OM) percentage can be expressed as Oxidisable Organic Carbon (OC) percentage as the conversion factor (also known as Van Bemmelen Factor and often taken as 1.72) from OC to OM has been reported to have varied depending upon soil type (Jain *et al.,* 1997) and soil depth (Westman *et al.,* 2006).
E. We will be grateful if instead of detailed writing; values and parameters mentioned under *Soil* subheading of **Materials and Method**; could have been arranged under a table and necessary brief description after the table for better comprehension along with *bibliographic references* of Soil Quality Indices (SQI) mentioned therein. For example –

| Initial Soil Parameters(XXX Site) | Safe limit/range | Current Result | Remarks | Reference/s for SQI |
| --- | --- | --- | --- | --- |
| pH |  |  |  |  |
| OC% |  |  |  |  |
| Initial Soil Parameters(YYY Site) | Safe limit/range | Current Result | Remarks | Reference/s for SQI |
| pH |  |  |  |  |
| OC% |  |  |  |  |

…etc.

Also we will be grateful for explanation of why Total fraction of soil N is estimated whereas only available fractions of P and K were estimated in the **Materials and Method**.

F. It is interesting to notice that the trials have been executed in three different year schedules for the three different zones. We would be grateful for the reason why the duration is 3years for Rampur site and 2years for the other two sites, as many factors that are evident to affect nutrient availability may vary with time. Please also mention the duration of experiment for each site *along with cropping cycle for each year* in the **Materials and Methods**.
G. We would be curious to know the reason behind similar Nitrogen (100 kg.ha^-1^) and Phosphorous (30 kg.ha^-1^) doses and split does techniques for both rice and wheat as each of them have different requirements that also varies with soil type and crop cultivation strategies. Please also mention the cultivation practice (like wetland paddy. arable rice cultivation/Terrace Farming/ SRI technique etc…) followed for rice in each region as Potassium movement has been observed to have affected according to periodicity of water regimes in rice-wheat system (Kadrekar and Kibe, 1973).
H. It would be appreciable if the references for protocols that have been used in analyzing soil parameters could have been mentioned in the **Materials and Methodology**. Please also mention the method of fertilizer application as Potassium movement in soil is reported to have affected by method of application (Nolan and Pritchett, 1960).
I. We will be grateful if you kindly flag (with ^*^ or ^**^ at superscript) the correlation coefficients in the text and in supplementary tables according to their significance and mention the version of M.S. Excel and R-software along with R-studio.
J. It would be appreciable if the term “**significantly different**” can be replaced with “**significantly increased/ decreased**”, wherever applicable in the text to be more specific. Also, it will be a bit easier to comprehend if before conclusion, the discussion section can be subdivided into three parts, discussing reasons behind the results for each of the areas separately and lastly another part dedicated to discussion for main and important aspects that has come out of the discussion of those three areas.
K. Soil pH is often considered as one of the important factors in determining nutrient availability in soil. Martin *et al.* (1946) showed at pH values up to 2.5 there was no K fixation, may be due to increased abundance of H_3_O^+^ that replace K from the exchange site (Rich and Black, 1964). However, *between pH 2.5 and 5.5*, the amount of K fixation increased very rapidly. Above pH 5.5, fixation increased more slowly (Martin *et al.*, 1946). As most of the soils of all three areas come under this study come within this pH 5.5 (Table S1), post application of potassium fertilizers, we will be grateful if some discussions can be added on how soil pH has affected the K availability in this study with specifications of sites.
L. In this context, soil pH of the Kabre, under Hilly area is quite low for rice cultivation than soil pH 5.0-6.5 for fertile wetland rice soil as mentioned by Dr. F. N. Ponnamperuma, the celebrated Rice Soil Chemist and former Principal Soil Chemist, International Rice Research Institute (IRRI), Los Banos, Philippines (Ponnamperuma, 1981). As most of the soil functions and microbial nutrient transformations get restricted below 5.5 (Pietri and Brooks, 2008), we will be grateful if this can be emphasized while discussing results of this area.

[ *Dr. F. N. Ponnamperuma, the celebrated Rice Soil Chemist and former Principal Soil Chemist, International Rice Research Institute (IRRI), Los Banos, Philippines listed the following following chemical characteristics of fertile wetland rice soils:*

*pH 5.0-6.5, ECe (mS/cm) <2, Eh (after submergence, in volt) +0.2 to -0.2, org matter (%) 2.0-3.5, total N (%) >0.2, total P (%) >0.02, Olsen P (mg/kg) >10, exch K (mmol/kg) >2, avail S (mg/kg) >10, CEC (mmol/kg) >200, clay composition >50% montmorillonite, active Fe (%) >0.5%, active Mn (%) >0.05, avail Zn (mg/kg) >1, avail B (mg/kg) <5.* ]

M. Soil Nitrogen and Phosphorus are also known to affect Potassium availability and uptake by plants. Application of nitrogen and phosphorus are reported to have resulted in 145% increase in potassium uptake as compared to control (Tandon and Sekhon, 1988). Tiwari *et al*. (1992) reported that response to potassium application in rice increased with increasing rate of nitrogen application. Under nitrogenous fertilization, interlayer potassium (K) can also be replaced by NH_4_^+^ as both of them have nearly similar ionic size, influencing its availability. Therefore, it will be appreciable if changes in these factors can be considered while discussing K availability with respect to control in this study.
N. We would appreciate if relations of Plant parameters (*yield influencing parameters with yield*) with changes in Potassium dosage applied in soil can be discussed in **Discussion** segment (like how plant height and yield can have a strong positive correlation in terms of changes in potassium content in soil; as revealed in the **Result** section.)
O. In the discussion section there is a mention “*Comparing the K fertilizer consumption under RWCS in the IGP regions, Nepal uses less 324 amount of potassium fertilizer in rice (0.8 kg ha-1) and wheat (1.7 kg ha-1) compared to China in rice (33.2 kg ha-1) and wheat (26.6 kg ha-1).*” It is quite unclear here whether the comparison is stated with China, or with IGP. We will be grateful if this could be explained properly. Also mention the region of IGP in bracket wherever the term IGP is mentioned, as IGP is a widespread region stretched amongst countries in **Indian subcontinent**.
P. We will be grateful if you can justify the term “Revised” in the title of the manuscript; in terms of how this *revision* is applicable to the whole country of Nepal as these trials are mentioned to have conducted in *localized research stations* and seem not to have checked in *selected progressive farmer’s field* with farmer’s packages of practices *throughout the country*. Also the selected trial sites mainly represent the *Central Development Region of Nepal* ("Memorial Step of King Mahendra in 1st Poush 2017 BS". reviewnepal.com. 13 December 2017. Retrieved 6 February 2018.), consisting of Province No. 2 (9,661 km²) and Bagmati Pradesh province (20,300 km²) which together share only 20.30% approx. of total land area (147,557 km²) of Nepal. This may have lack of implacability of these doses to other areas of Nepal (distant from these research stations), which may vary in terms of soil-water and land use characteristics. .
Q. It is a pleasure to observe that in the Manuscript; only 11% similarity has been found on Plagiarism Checker X 2018 Professional Edition v6.0.6. (Report Attached). We will be grateful if this can be improved even more

**References:**

J. C. A. Pietri and P. C. Brookes, “Relationships between soil pH and microbial properties in a UK arable soil,” Soil Biology & Biochemistry, vol. 40, no. 7, pp. 1856–1861, 2008.

Jain, T. B., Graham, R. T. and Adams, D. L. 1997. Carbon to organic matter ratios for soils in Rocky Mountain coniferous forests. Soil Sci. Am. J. 61: 11901195.

Kadrekar, S.B. and Kibe, M. M. (1973). Release of soil potassium on wetting and drying*. J. Indian Soc. Soil Sci*., 20:231-240.

Martin, J.C., Overstreet, R. and Hoagland, D.R. 1946. Potassium fixation in soils in replaceable and nonreplaceable forms in relation to chemical reactions in the soil. Soil Science Society of America Proceedings 10: 94-101.

Nolan, C.N. and Pritchett, W.L. 1960. Certain factors affecting the leaching of potassium from sandy soils. Proceedings of Soil Crop Science Society of Florida 20: 139-145.

Pannu, R.P.S., Singh, Y., Singh, B. (2003). Effect of long term application of organic materials and inorganic N-fertilizer on potassium fixation and release characteristics of soils under rice-wheat cropping system. *J. Potassium Res.* (in press)

Ponnamperuma, F.N. (1981). Properties of Tropical Rice Soils. Text of a Series of Lectures Delivery to Graduate Students at the Topical Agriculture College, H. Cardenas, Tabasco, Mexico on 23-25 July 1981.

Rich, C.I., and Black, W.R. 1964. Potassium exchange as affected by cation size, pH, and mineral structure. Soil Science 97: 384-390.

Tandon, H.L.S. and Sekhon, G.S. 1988. Potassium Research and Agricultural Production in India. 144 pp. Fertiliser Development and Consultation Organization, New Delhi.

Tiwari, K.N., Dwivedi, B.S. and Subbarao, A. 1992. Potassium management in rice-wheat system, pp. 93-114. In Pandey, R.K., Dwivedi, B.S. and Sharma, A.K. (eds.) Rice-Wheat Cropping System: Proceedings of Rice-Wheat Workshop, Project Directorate for Cropping Systems Research. Modipuram, India.

Westman, C. J., Hyto¨ nen, J. and Wall, A. 2006. Loss-onignition in the detremination of pools of organic carbon in soils of forests and afforested arable fields. Commun. Soil Sci. Plant Anal. 37: 10591075.
