# Supplementary material for: Potassium nutrient response in the rice-wheat cropping system in different agro-ecozones of Nepal
Source: PLoS One. 2021 Mar 18;16(3):e0248837. doi: 10.1371/journal.pone.0248837 (PMC7971854; doi:10.1371/journal.pone.0248837)
Supplement: Attachment — Submitted filename: Reviewers comments.docx [file pone.0248837.s004.docx]

The authors Ojha et al. have applied different rates of K fertilizer to estimate crop response to the added K in three agro-ecozones in Nepal where soils are K limited. They used the K rates as K2O, which form of K compound is absent in soil, in reality. The findings do not provide with ground breaking information in K chemistry in soils nor provide with any significant knowledge gaps in K fertilization in crops. It is quite known that in a K limited soil, added K will increase crop production. The statistical method for estimating the optimum K rate is not as robust. As an international and high ranked journal, authors need to provide K uptake, balance and surplus data in your study sites. However, the information bears some merits within the scope of publication in PLOSE ONE. Yet, there are some major flaws in all sections which need to be addressed before it is accepted for publication in PLOSE ONE. My specific comments are added below:

Abstract

Please convert K2O to K and use consistently throughout the paper

Define RDKF L30

What is your hypothesis to use a wide range of K? Did the K content varies that wide?

Mention the duration of work in the abstract L35

L 39, you said maximum should not exceed, rather you should mention what would be the optimum rate for higher economic production

Four times higher than the current rate looks unusually high

Introduction

The aim was yield and nutrient response but nutrient is not only the K, Have you measured K concentrations in grain and straw

Methods

In this section authors should add some initial soil characteristics including K minerals in a table.

L108; define masl

Aadd appropriate references for the weather data given, if it is measured in your station, mention details of the methods of data recording

L137, define OM in its first use

It is better to provide soil quality parameters in a table

Explain the soil sampling time and strategies more details.

Results

Why did you record 1000 grain weight, plant height and panicle length, you have to show the significance of recording these data on the grain and straw yields.

As an international and high ranked journal, you need to provide K uptake, balance and surplus data in your study sites

L187; You evaluated soil properties in different years in different sites which definitely had different climatic conditions, especially rainfall.

L199; where are the graphical presentations of the trend?

You showed yield data from 2009 to up until 2014, but not uniform in different sites?

Table 5; these analysis are based on one year or three years data?

L287; it is not clear in your paper about how did you estimate the residual fertility and its effect on grain yield.

Discussion

L302; this is not clear about what should be the exact K rate

L305, you said about nutrient but should specifically say about K, because you are not estimating other nutrients

L309; witnessed is not appropriate wording here

319; 15 kg what?

L324; potassium, please use K

L327; please update the rates used in Bangladesh etc. it looks much lower than the current rates

overall the discussion doesn’t link very well with your findings.
