# Supplementary material for: Potassium nutrient response in the rice-wheat cropping system in different agro-ecozones of Nepal
Source: PLoS One. 2021 Mar 18;16(3):e0248837. doi: 10.1371/journal.pone.0248837 (PMC7971854; doi:10.1371/journal.pone.0248837)
Supplement: Attachment — Submitted filename: Revised Review Notes_Nepal.docx [file pone.0248837.s006.docx]

We are grateful to receive necessary modifications of the manuscript entitled “Revised potassium nutrition in rice-wheat cropping system of Nepal” by Ojha *et. el.* Although the authors have employed considerable efforts for the improvement, we have observed some points from the previous suggestions have not been addressed properly. We would appreciate if the authors could consider the previous ones as well as the new ones as follows to reconstruct the manuscript.

1. We understand that in the abstract section the authors described a mean response of Potassium fertilization, irrespective of rice/wheat cultivated along with doses for economic optimum. However, a mean fertilizer response value for two different crops, **having different potassium requirement (**[**http://www.fao.org/3/a0443e/a0443e04.pdf**](http://www.fao.org/3/a0443e/a0443e04.pdf) **)**, may still create confusion. It would be, therefore, appreciable that the authors avoid the ‘mean response’ part from the abstract **as well as from the result/discussion section.**
2. We appreciate the calculation of “Economic optimum” and would be grateful to receive a **bibliographic reference** in support of the calculation of “Economic optimum”.
3. In Materials and Methods, winter season has still been mentioned as “…generally cold and humid with scarce rainfall.” We would appreciate if authors could mention that how “Humidity” can be related with “Scarcity of rainfall”?
4. We are grateful for the sincere effort of the authors, applied for construction of Table-1. However, we feel that there may be lack of comprehensibility in the previous suggestion, that the Van Bemmelen Factor (often taken as 1.72) varies with soil type (Jain *et al.*, 1997) and soil depth (Westman *et al.*, 2006), **leading to varying results of OM depending upon soil type and depth**. It is, therefore highly appreciable to express the OM content as OC throughout the manuscript. Regarding Total soil Nitrogen analysis, we will be grateful to have **bibliographic mention** of the recommendation of the “Central Soil Laboratory” procedures of soil analysis. Please also provide bibliographic references to the analytical protocols (like Olsen, Walkley-Black etc…) mentioned.
5. In table number 5, one of the data series under column “straw yield” is mentioned to be recorded in 2012. Please rectify the ‘year’ if this is a flaw or else explain.
6. Regarding suggestion tagged under “G”, we appreciate that the cultivation practices of rice and wheat is mentioned in the text. However, we are still curious to know why similar dose and split dose techniques for Nitrogen (100 kg.ha^-1^) and Phosphorous (30 kg.ha^-1^) were applied for both rice and wheat as each one of them have different nutrient requirements (<http://www.fao.org/3/a0443e/a0443e04.pdf> ). Please address this query.
7. Please mention table number and/or figure number along with respective findings (value of economic optimum, K doses and yield parameters) in the Discussion section.
8. It is quite appreciable that the authors modified the discussion part. However, a major portion of the discussion still engages in establishing “Why” the additional dose of Potassium may be useful, whereas, it misses “How” it may be useful, to a great extent. Please address point “N” from the previous suggestion more clearly and relate the findings with applied potassium dosages (like why there is a 30-33% rice yield decline in Rampur site in 2^nd^ year? Why there is a 38% grain yield and 20% straw yield decline over 3 years in Rampur site? **How applied potassium influenced all these increase and/or decrease in all these three experimental sites**? What role potassium is playing in soil and/or plant body to increase and/or decrease yield in all these three sites specifically? Please relate these kinds of major findings with applied potassium and include in the discussion portion).
9. Please reconstruct the conclusion part mentioning Potassium recommendation according to economic optimum for both Rice and Wheat for all three experimental regions. It is highly advisable to mention those recommendations according to the crops and varieties put under experimental trials instead of mentioning to be fruitful for any other hybrid varieties not tested in this experiment. Please also rectify this in the Discussion part as well.
10. Regarding point “F” of our previous queries, we will be grateful to receive a more clear response on why the duration is 3 years for Rampur site and One year less (2 years) for the other two sites. Please mention crop cycles in detail (each year; each site), crops cultivated in those fields and fertilizers previously used.
11. We understand and appreciate the way the authors mentioned limitations of this study in the text. However, we feel that the mentions of limitations of research itself in the text may question the relevance of the research hypothesis. Please express the limitations as **Future scope/s of Research** and avoid words like “pitfall”, “limitations” etc. And, since it is not very convincing to declare the resulted dose to be **revised** without being rigorously tested in farmer’s fields (*where soil characteristics may differ from the research stations depending upon farmer’s packages of cultivation practices and crop cycles*) of the representative agro-eco zones of Nepal, we will be grateful, instead, if the authors could modify the title, by including, what this research is basically aimed at according to the objectives. [ like “*Effect of increased dose of Potassium over recommendation on yield and nutrient response of rice and wheat in High hill, Inner-terai and Terai region of Nepal*” or whichever is convenient.]
12. We really appreciate the hard work of the authors employed on reducing plagiarism of this text from 11% to 9% as found on Plagiarism Checker X 2018 Professional Edition v6.0.6. (Report attached).

References:

Jain, T. B., Graham, R. T. and Adams, D. L. 1997. Carbon to organic matter ratios for soils in Rocky Mountain coniferousforests. Soil Sci. Am. J. 61: 11901195.

Westman, C. J., Hyto¨ nen, J. and Wall, A. 2006.Loss-onignitionin the detremination of pools of organic carbon insoils of forests and afforested arable fields.Commun. Soil Sci.Plant Anal. 37: 10591075.
