# Supplementary material for: Potassium nutrient response in the rice-wheat cropping system in different agro-ecozones of Nepal
Source: PLoS One. 2021 Mar 18;16(3):e0248837. doi: 10.1371/journal.pone.0248837 (PMC7971854; doi:10.1371/journal.pone.0248837)
Supplement: Attachment — Submitted filename: 3. Revised Review_Response.docx [file pone.0248837.s008.docx]

We are grateful to receive necessary modifications of the manuscript entitled “Revised potassium nutrition in rice-wheat cropping system of Nepal” by Ojha *et. el.* Although the authors have employed considerable efforts for the improvement, we have observed some points from the previous suggestions have not been addressed properly. We would appreciate if the authors could consider the previous ones as well as the new ones as follows to reconstruct the manuscript.

***[Response]****: We would like to thank you very much for this second round review and your effort to make our work a standard quality. We are trying to address each and every query in details.*

We understand that in the abstract section the authors described a mean response of Potassium fertilization, irrespective of rice/wheat cultivated along with doses for economic optimum. However, a mean fertilizer response value for two different crops, **having different potassium requirement (**[**http://www.fao.org/3/a0443e/a0443e04.pdf**](http://www.fao.org/3/a0443e/a0443e04.pdf) **)**, may still create confusion. It would be, therefore, appreciable that the authors avoid the ‘mean response’ part from the abstract **as well as from the result/discussion section.**

***[Response]****: We thank you for your valuable comment and suggestion. The pdf file was helpful to clarify our concept. We have removed the ‘mean response’ word from all the parts of the manuscript accordingly.*

1. We appreciate the calculation of “Economic optimum” and would be grateful to receive a **bibliographic reference** in support of the calculation of “Economic optimum”.

***[Response]****: We have provided the reference [40] as a reference to calculate the Economic optimum and the reference is provided.*

2. In Materials and Methods, winter season has still been mentioned as “…generally cold and humid with scarce rainfall.” We would appreciate if authors could mention that how “Humidity” can be related with “Scarcity of rainfall”?

***[Response]:*** *Yes, we agree with your argument. However, several literatures referred winter as cold and humid. In fact, we received low temp (<10 ^0^C) and high relative humidity (>90-95%). Rainfall is scarce compared to monsoon but is enough to maintain the humidity. So, we think it is clear. To avoid confusion, we have removed ‘scarce rainfall’ from that sentence.*

3. We are grateful for the sincere effort of the authors, applied for construction of Table-1. However, we feel that there may be lack of comprehensibility in the previous suggestion, that the Van Bemmelen Factor (often taken as 1.72) varies with soil type (Jain *et al.*, 1997) and soil depth (Westman *et al.*, 2006), **leading to varying results of OM depending upon soil type and depth**. It is, therefore highly appreciable to express the OM content as OC throughout the manuscript. Regarding Total soil Nitrogen analysis, we will be grateful to have **bibliographic mention** of the recommendation of the “Central Soil Laboratory” procedures of soil analysis. Please also provide bibliographic references to the analytical protocols (like Olsen, Walkley-Black etc…) mentioned.

***[Response]:*** *We have changed OM to OC throughout the manuscript as per your suggestion. We also have provided the bibliographic references of the analytical protocols.*

4. In table number 5, one of the data series under column “straw yield” is mentioned to be recorded in 2012. Please rectify the ‘year’ if this is a flaw or else explain.

***[Response]:*** *We are very grateful to the reviewer to spot this kind of detail review. Thank you very much. We have corrected it.*

5. Regarding suggestion tagged under “G”, we appreciate that the cultivation practices of rice and wheat is mentioned in the text. However, we are still curious to know why similar dose and split dose techniques for Nitrogen (100 kg.ha^-1^) and Phosphorous (30 kg.ha^-1^) were applied for both rice and wheat as each one of them have different nutrient requirements (<http://www.fao.org/3/a0443e/a0443e04.pdf> ). Please address this query.

***[Response]****: We again thank you for repeating this query about N and P dose and application method similar for rice and wheat. This is the blanket national recommendation for rice and wheat and we have followed the same recommendation. As you see the K rate is also recommended the same for rice and wheat. Our objective was to recommend a revised K rate in place of this blanket K rate. Our finding will be valuable to convince our policy maker to make a separate and robust recommendation in each agro-zone and in each crop type.*

6. Please mention table number and/or figure number along with respective findings (value of economic optimum, K doses and yield parameters) in the Discussion section.

***[Response]****: We added the table and figure reference in the respective findings of the discussion section.*

7. It is quite appreciable that the authors modified the discussion part. However, a major portion of the discussion still engages in establishing “Why” the additional dose of Potassium may be useful, whereas, it misses “How” it may be useful, to a great extent. Please address point “N” from the previous suggestion more clearly and relate the findings with applied potassium dosages (like why there is a 30-33% rice yield decline in Rampur site in 2^nd^ year? Why there is a 38% grain yield and 20% straw yield decline over 3 years in Rampur site? **How applied potassium influenced all these increase and/or decrease in all these three experimental sites**? What role potassium is playing in soil and/or plant body to increase and/or decrease yield in all these three sites specifically? Please relate these kinds of major findings with applied potassium and include in the discussion portion).

***[Response]****: You are right, how additional doses of K fertilizer is used by produced biomass or grain can be included in Discussion section. However, it was our limitation (we did not run grain/ biomass K analysis) as well as out of scope (we focused on application of different K rates on crop yield and soil properties). Besides, in our Results section- 3.1: Soil chemical properties, we have mentioned that no significant changes in soil N concentration due to addition of different K rates. So, we were unable to discussion further about N and K relationship in Discussion section have already mentioned as follow-*

*L#386-388: The current study reported that there were no differences in soil total N after a year’s addition of K fertilizer, suggesting no adverse effects on total N due to treatment application.”*

*We added following sentence to make it clear—*

*L#388-390: “However, our study limits that we did not observe the effects of different rates of K fertilizer on uptake of nutrients from the soil by plant roots for grain yield and biomass production.”*

8. Please reconstruct the conclusion part mentioning Potassium recommendation according to economic optimum for both Rice and Wheat for all three experimental regions. It is highly advisable to mention those recommendations according to the crops and varieties put under experimental trials instead of mentioning to be fruitful for any other hybrid varieties not tested in this experiment. Please also rectify this in the Discussion part as well.

***[Response]****: We have added the potassium recommendation according to our economic optimum value. We also have removed the part of ‘high yielding and improved varieties” part from the conclusion. Yes, we believe it helps to put our recommendation in a stronger way.*

9. Regarding point “F” of our previous queries, we will be grateful to receive a more clear response on why the duration is 3 years for Rampur site and One year less (2 years) for the other two sites. Please mention crop cycles in detail (each year; each site), crops cultivated in those fields and fertilizers previously used.

***[Response]****: There is no typical objective to take 3 years measurement from Rampur site and 2 years measurement from other two sites. Rampur site is located in accessible area, so a three year harvest is possible. Other two sites, only 2 years measurement is possible due to lack of accessibility. We are trying to be honest to put all the data from all sites that we have taken during the research period. We understand the concern of reviewer that three year data is more reliable but we hope you will understand and accept our condition. We thought two year data is enough to justify our objective to monitor the crop response with additional K rate. As our objective is not to compare between the locations, this year difference doesn’t affect our statistical analysis.*

10. We understand and appreciate the way the authors mentioned limitations of this study in the text. However, we feel that the mentions of limitations of research itself in the text may question the relevance of the research hypothesis. Please express the limitations as **Future scope/s of Research** and avoid words like “pitfall”, “limitations” etc. And, since it is not very convincing to declare the resulted dose to be **revised** without being rigorously tested in farmer’s fields (*where soil characteristics may differ from the research stations depending upon farmer’s packages of cultivation practices and crop cycles*) of the representative agro-eco zones of Nepal, we will be grateful, instead, if the authors could modify the title, by including, what this research is basically aimed at according to the objectives. [ like “*Effect of increased dose of Potassium over recommendation on yield and nutrient response of rice and wheat in High hill, Inner-terai and Terai region of Nepal*” or whichever is convenient.]

***[Response]****:*

*We fully agree with the reviewer, the word ‘pitfall’ reduce the strength of our work. So, we have removed it in the revised manuscript. Now, we realise it provides a great sense. Thank you very much.*

*Our objective is to recommend a new rate of K dose specific to the agro-ecozones in rice and wheat cropping system. Yes, definitely rigorous field trial is necessary but we think “Revision of potassium” is right keyword in the topic. Yes, we definitely agree, the word ‘nutrition’ is not suitable as you suggested in the previous comment (8). We are decided to change it as ‘rate’. And of course it is representative of “Terai, inner-terai, and high-hills” which you have suggested to include it as well. We replaced it with word “agro-ecozones” to shorten the title word count. So, our revised title is “Revision of potassium rate in rice-wheat cropping system in three agro-ecozones of Nepal”.*

11. We really appreciate the hard work of the authors employed on reducing plagiarism of this text from 11% to 9% as found on Plagiarism Checker X 2018 Professional Edition v6.0.6. (Report attached).

***[Response]****: Thank you very much for checking this one of the important publication integrity components. We highly appreciate your sincere review.*

References:

Jain, T. B., Graham, R. T. and Adams, D. L. 1997. Carbon to organic matter ratios for soils in Rocky Mountain coniferousforests. Soil Sci. Am. J. 61: 11901195.

Westman, C. J., Hyto¨ nen, J. and Wall, A. 2006.Loss-onignitionin the detremination of pools of organic carbon insoils of forests and afforested arable fields.Commun. Soil Sci.Plant Anal. 37: 10591075.
