# Supplementary material for: Potassium nutrient response in the rice-wheat cropping system in different agro-ecozones of Nepal
Source: PLoS One. 2021 Mar 18;16(3):e0248837. doi: 10.1371/journal.pone.0248837 (PMC7971854; doi:10.1371/journal.pone.0248837)
Supplement: Attachment — Submitted filename: 3rd Review Notes_Potassium.docx [file pone.0248837.s009.docx]

We appreciate constant and sincere effort of the authors in making the revised manuscript better. However, in case of some responses made by the authors, it is hard for us to agree. These are as follows-

1. Regarding the response of point 10, we would like to mention that we have never suggested the term “nutrition” to be ‘not suitable’ in our comment number 8. Instead we mentioned that we would prefer if the authors include the terms “nutrient response” and “yield response” into the title. We urge the authors to be cautious on making such acquisitions.

We understand the author’s concern that a revised recommendation will help in making accurate policies in the country. However, as mentioned earlier, it is not only unscientific, but also unethical to declare dose/s of potassium as revised for the entire country based on experimental research in only 3 research stations, without considering being crossed checked the results in farmer’s field even for once! Rigorous field test is a must for any recommendation.

Furthermore, Potassium, being an active cation, also influences dynamics of other nutrient ions in soil as well as in plant body. For example, Potassium indirectly influences B availability due to its effect on Ca absorption (Mattigod *et al.* 1985) and it is well known that a very narrow range of deficiency and toxicity of boron in plant body can severely affect the yield. This is just one example… there could be many more!

As mentioned in response of point 7, the authors have provided no robust explanations in discussions of their own findings (like why there is a 30-33% rice yield decline in Rampur site in 2nd year? Why there is a 38% grain yield and 20% straw yield decline over 3 years in Rampur site? Etc…). And as the researchers did not check for the dynamics and uptake of potassium content in plant parts, there is no direct evidence of how potassium influenced the yield in this experiment. The limitation of this work itself inhibits from calling it a ‘Revision’.

Therefore, we could not be more positive here for the publication of this article due to aforesaid reasons and absence of the author’s effort in modifying these key concerns even after two revision attempts and we would like to rely upon the respected Editors for the final decision.

We really appreciate and would like to thank the authors for the other necessary modifications made so far. Although this manuscript has some merit regarding positive responses of potassium application on rice and wheat; the authors should be more considerate in segregating research hypothesis in context with the unexplained portions from result and discussion segment and need to reconstruct the title according to the objectives achieved through this research.

Some more points that could be taken under consideration in future are as follows-

2. Bibliographic reference of “Economic optimum” as provided by the authors is not robust enough to understand the application of this procedure in the present context. We have downloaded and read the book which includes some implications of “economic optimum” regarding efficiency of nitrogen application. We would appreciate if the authors could provide a reference where the concept of “economic optimum” is well explained.
3. Please provide full form and bibliographic reference of SSD (line 158). Please also mention in the text and provide reference to the “National blanket recommendation” as mentioned in response to comment number 5.

**Reference:**

Mattigod SV, Frampton JA, Lim CH (1985) Effect of ion-pair formation on boron adsorption by kaolinite. Clay Clay Miner 33:433–437
