# Supplementary material for: Potassium nutrient response in the rice-wheat cropping system in different agro-ecozones of Nepal
Source: PLoS One. 2021 Mar 18;16(3):e0248837. doi: 10.1371/journal.pone.0248837 (PMC7971854; doi:10.1371/journal.pone.0248837)
Supplement: Attachment — Submitted filename: 3. Response_3rd Review Notes.docx [file pone.0248837.s010.docx]

We appreciate constant and sincere effort of the authors in making the revised manuscript better. However, in case of some responses made by the authors, it is hard for us to agree. These are as follows-

1. Regarding the response of point 10, we would like to mention that we have never suggested the term “nutrition” to be ‘not suitable’ in our comment number 8. Instead we mentioned that we would prefer if the authors include the terms “nutrient response” and “yield response” into the title. We urge the authors to be cautious on making such acquisitions.

[***Response***]: We would like to thank reviewer for their insightful review remarks. Please accept our sincere apologies for the inconvenience. We tried our best to address your concern in our manuscript. After careful and critical discussion among the authors, we have decided to change the title of the manuscript as per the reviewer’s suggestion. Here is the sequence of the manuscript title that we have changed with the progress of the review.

*Original title:* Revised potassium nutrition in rice-wheat cropping system of Nepal

*After first revision:* Revision of potassium rate in the rice-wheat cropping system in different agro-ecozones of Nepal.

*After second revision*: Potassium nutrient response in the rice-wheat cropping system in different agro-ecozones of Nepal.

So, we are grateful with the reviewer to re-shape our manuscript title that is justifiable to our work.

We understand the author’s concern that a revised recommendation will help in making accurate policies in the country. However, as mentioned earlier, it is not only unscientific, but also unethical to declare dose/s of potassium as revised for the entire country based on experimental research in only 3 research stations, without considering being crossed checked the results in farmer’s field even for once! Rigorous field test is a must for any recommendation.

[***Response***]: We now believe the revised manuscript title will address this concern of reviewer. We also have changed our argument in abstract and introduction to justify the manuscript title. We have removed the “a new fertilizer recommend rate” from the recommendation section of abstract (line 44), however, suggest to increase new K rate that and suggest to intensify this work in different locations (line 47-48). Hope this will address the reviewer’s concern in the right way.

Furthermore, Potassium, being an active cation, also influences dynamics of other nutrient ions in soil as well as in plant body. For example, Potassium indirectly influences B availability due to its effect on Ca absorption (Mattigod *et al.* 1985) and it is well known that a very narrow range of deficiency and toxicity of boron in plant body can severely affect the yield. This is just one example… there could be many more!

[***Response***]: Yes, we agree our data limits us to explain this influence. We have not witnessed any of such deficiency and toxicity symptoms in the field crop in any of the research plots assuming that they are not likely to influence our results.

As mentioned in response of point 7, the authors have provided no robust explanations in discussions of their own findings (like why there is a 30-33% rice yield decline in Rampur site in 2nd year? Why there is a 38% grain yield and 20% straw yield decline over 3 years in Rampur site? Etc…). And as the researchers did not check for the dynamics and uptake of potassium content in plant parts, there is no direct evidence of how potassium influenced the yield in this experiment. The limitation of this work itself inhibits from calling it a ‘Revision’.

[***Response***]: Overall, we see the yield increment with increasing potassium rate, so we focused our discussion on this point. We respect the reviewer’s concern. This decline is not following in the next year and the yield response is increasing with increased K rate. So, we concentrated discussing our point highlighting the importance of K increment for increased yield.

Yes, we accept our limitation of our study and the discussion points that we have incorporated in previous revised version (*recall*: line 403-405). After changing the title and hypothesis after this third revision we think this will justify the current motivation of the study.

Therefore, we could not be more positive here for the publication of this article due to aforesaid reasons and absence of the author’s effort in modifying these key concerns even after two revision attempts and we would like to rely upon the respected Editors for the final decision.

[***Response***]: We think we have addressed the concern of the reviewer by changing the title and motivation of our work along with changing the conclusion and last paragraph of the discussion section. We completely change the idea of ‘new K rate recommendation’ to ‘response of K fertilizer application’ that makes the whole essence of this manuscript in a way more logical than previous version. We will always be thankful to the reviewers for their insightful comments and suggestions.

We came up with a very long way to make this manuscript to this publication stage. We believe the result and recommendation of this paper will be an important testimony to make national K fertilizer management plan and provides a strong support for conducting further field verification trials. So, we are very much hopeful with reviewer and editor to consider this revised manuscript for the publication.

We really appreciate and would like to thank the authors for the other necessary modifications made so far. Although this manuscript has some merit regarding positive responses of potassium application on rice and wheat; the authors should be more considerate in segregating research hypothesis in context with the unexplained portions from result and discussion segment and need to reconstruct the title according to the objectives achieved through this research.

[***Response***]: We have reconstructed the title, objective and hypothesis (last paragraph of the introduction, line 98-104) of our research which address the limitation of our work raised by reviewer. We would like to thank reviewer for this great suggestion.

Some more points that could be taken under consideration in future are as follows-

2. Bibliographic reference of “Economic optimum” as provided by the authors is not robust enough to understand the application of this procedure in the present context. We have downloaded and read the book which includes some implications of “economic optimum” regarding efficiency of nitrogen application. We would appreciate if the authors could provide a reference where the concept of “economic optimum” is well explained.

[***Response***]: We have added the reference of the ‘economic optimum’ concept in the revised manuscript (line 204).

3. Please provide full form and bibliographic reference of SSD (line 158). Please also mention in the text and provide reference to the “National blanket recommendation” as mentioned in response to comment number 5.

[***Response***]: We have added the details of bibliographic reference as [36] in line 163.

We also have added the reference of national blanket fertilizer recommendation (line 181).

**Reference:**

Mattigod SV, Frampton JA, Lim CH (1985) Effect of ion-pair formation on boron adsorption by kaolinite. Clay Clay Miner 33:433–437
